# Enhancing Biomolecular Simulations with Hybrid Potentials Incorporating NMR Data

**DOI:** 10.1021/acs.jctc.2c00657

**Published:** 2022-11-17

**Authors:** Guowei Qi, Michail D. Vrettas, Carmen Biancaniello, Maximo Sanz-Hernandez, Conor T. Cafolla, John W. R. Morgan, Yifei Wang, Alfonso De Simone, David J. Wales

**Affiliations:** † Department of Chemistry, University of Cambridge, Lensfield Road, CambridgeCB2 1EW, U.K.; ‡ Department of Pharmacy, University of Naples Federico II, 80131Naples, Italy; § Department of Life Sciences, Imperial College London, South Kensington, LondonSW7 2AZ, U.K.

## Abstract

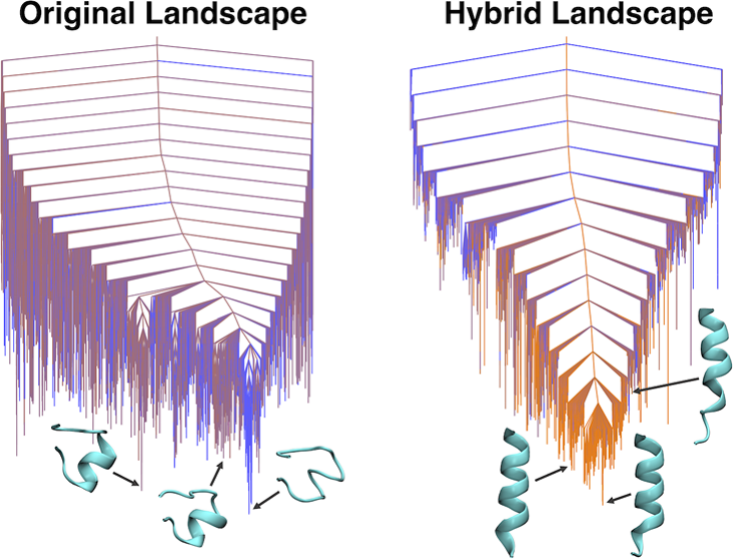

Some recent advances in biomolecular simulation and global optimization have used hybrid restraint potentials, where harmonic restraints that penalize conformations inconsistent with experimental data are combined with molecular mechanics force fields. These hybrid potentials can be used to improve the performance of molecular dynamics, structure prediction, energy landscape sampling, and other computational methods that rely on the accuracy of the underlying force field. Here, we develop a hybrid restraint potential based on NapShift, an artificial neural network trained to predict protein nuclear magnetic resonance (NMR) chemical shifts from sequence and structure. In addition to providing accurate predictions of experimental chemical shifts, NapShift is fully differentiable with respect to atomic coordinates, which allows us to use it for structural refinement. By employing NapShift to predict chemical shifts from the protein conformation at each simulation step, we can compute an energy penalty and the corresponding hybrid restraint forces based on the difference between the predicted values and the experimental chemical shifts. The performance of the hybrid restraint potential was benchmarked using both basin-hopping global optimization and molecular dynamics simulations. In each case, the NapShift hybrid potential improved the accuracy, leading to better structure prediction via basin-hopping and increased local stability in molecular dynamics simulations. Our results suggest that neural network hybrid potentials based on NMR observables can enhance a broad range of molecular simulation methods, and the prediction accuracy will improve as more experimental training data become available.

## I Introduction

Natural proteins that perform a single function have evolved to possess a funneled energy landscape (EL), enabling them to fold into functional native states that underlie biological activity.^1,2^ Alternatively, the EL can exhibit multiple funnels associated with multiple functions such as catalysis, protein–protein interactions, self-assembly, and transport through a cell membrane. The variety of thermally accessible conformations is significantly enhanced in the case of intrinsically disordered proteins (IDPs), characterized by multiple funnels, which we have suggested may correlate with multiple functions.^3^

In the past decades, significant progress has been made in the characterization of protein structures by three complementary techniques of structural biology: X-ray crystallography, nuclear magnetic resonance (NMR) spectroscopy, and cryogenic electron microscopy (cryo-EM). However, most of the current approaches are generally limited to well-defined and relatively rigid native states. The refinement of atomic structures using these techniques has limitations when analyzing proteins featuring structural flexibility and conformational heterogeneity. Hence, computational methods involving EL sampling and molecular simulations have an important role to play in studying the dynamics of flexible proteins. These simulation methods rely on the accuracy of empirical force fields, where intra- and interatomic potentials are modeled as physics-based functional forms. Force fields are refined using a combination of prior chemical knowledge and experimental results, leading to parameters that can reproduce experiments via computer simulations. However, these force fields are inherently limited in accuracy, as approximations to atomic interactions are often made for the sake of reducing computational expense.

A successful approach to improving biomolecular simulations involves the definition of hybrid potentials, where empirical force fields are combined with experimental data to achieve more accurate representations of protein structure and dynamics.^4,5^ Hybrid potentials for molecular simulations have been developed using different types of experimental data, with NMR playing a primary role. NMR experiments generate observables that cover a variety of timescales, probing strong stable and weak transient interactions, and reporting on local and global structural parameters in biomolecules. The most generally accessible parameter in NMR is the chemical shift (CS). The CS provides key experimental information on the structure and dynamics of proteins regardless of their conformational nature, including globular proteins, IDPs, amyloids, and membrane proteins. Successful methods have been designed to generate *ab initio* structures of proteins using CS, including CS-Rosetta^4^ and Almost,^6^ and several programs also exist to calculate protein CS from structure.^7−9^

NMR CS restraint potentials, together with molecular mechanics force fields, have previously been shown to improve native protein structure prediction using Monte Carlo,^10^ molecular dynamics,^11^ and basin-hopping (BH) global optimization^12^ simulations. These studies used polynomial functions of interatomic distances to generate a physics-based prediction of chemical shifts given a protein structure. Since the functions are fully differentiable, forces can be derived from the computed restraint potential and applied in simulations.

In the current work, we pair a molecular mechanics force field with an experimental hybrid restraint potential based on NapShift, a new machine learning model that predicts backbone chemical shifts from protein structure. NapShift uses protein sequence and dihedral angles as inputs to a neural network and outputs chemical shifts for each backbone atom in the protein. Differentiating the energy penalty between the calculated CS and the experimental results with respect to the atomic coordinates gives us a restraint force to pair with an empirical force field.

To analyze the changes in the underlying energy landscape introduced by a restraint potential, we employed two small benchmark protein systems: tryptophan zipper 1 (PDB^13^ code 1LE0(14)), a 12-residue peptide that folds into a β-hairpin with specific packing of tryptophan side chains, and a 15-residue peptide based on the sequence of residues 56 to 70 of human platelet factor 4 (PDB code 1DN3(15)), which was experimentally shown to form a stable α-helix in sodium dodecyl sulfate (SDS). We used a combination of BH global optimization^16−18^ and discrete path sampling^19−21^ to sample the energy landscape and search for the native folded structure of each protein, using both the original molecular mechanics force field and the hybrid force field, with varying contributions from the NMR restraint potential.

After benchmarking these two initial systems, we explored a more challenging test case, namely, an 18-residue peptide (DP5^22^) designed by Araki and Tamura to exhibit equal propensities for the α-helical and β-hairpin forms. Using calculated NMR restraints, we guided BH global optimization simulations toward either the α-helix (PDB code 2DX3) or the β-hairpin (PDB code 2DX4) to explore the effect that the hybrid potential has on a multifunnel energy landscape.

To determine the effectiveness of hybrid restraints on larger globular proteins, we used the NapShift hybrid potential for BH global optimization of ubiquitin. Ubiquitin has been experimentally^23^ and computationally^24^ shown to transition between two distinct conformations: the native conformation (Ub) and a sparsely populated conformation, where the C-terminal tail is retracted (Ub-CR). Using both the original force field and the hybrid potential, we attempted to locate the native Ub conformation by starting a BH global optimization run from the Ub-CR conformation.

We also incorporated this methodology into the GROMACS^25^ software for molecular dynamics (MD) simulations. We found significant gains in accuracy for MD simulations with a NapShift hybrid potential for the tryptophan zipper 1 as well as for ubiquitin with orthogonal NMR data.

## II Methods

### A. Datasets

NapShift was parameterized using a set of 3237 protein entries for which PDB structures and Biological Magnetic Resonance Data Bank^26^ (BMRB) CS are known. PDB files containing multiple structural models were treated by considering only the first model.

Individual CS values showing more than 5% relative error, calculated as ϵ_rel_ = |ϵ|/|*x*| (where |ϵ| = |*x* − *x*_approximate_| is the absolute error and *x* ≠ 0), were excluded from the training set. This protein database was decomposed into 2987 entries for training and validation, and 250 entries for testing the accuracy of the method (see Tables S3 and S4 in the Supporting Information for the PDB structures employed in this work.).

### B. Artificial Neural Network Modeling of CS

To predict CS values of the protein backbone atoms N, C, Cα, Cβ, H, and Hα, we developed simple feed-forward, fully connected, artificial neural networks^27^ (ANNs), and trained them using structural parameters and sequence information derived from PDBs. Each target atom was treated independently from the others, requiring six independent ANNs.

#### 1. Neural Network Architecture

To obtain a model that was differentiable with respect to Cartesian coordinates, we based our ANN architecture exclusively on the torsion angles of the protein, namely, ϕ, ψ, χ_1_, and χ_2_. Compared to more complex architectures, such as SPARTA+,^8^ we excluded some parameters, such as hydrogen bonds and ring current effects. In our tests, these additional terms provided small improvements in CS predictions, but they added significant complexity in defining Cartesian derivatives, which we wished to avoid.

**Figure 1:**
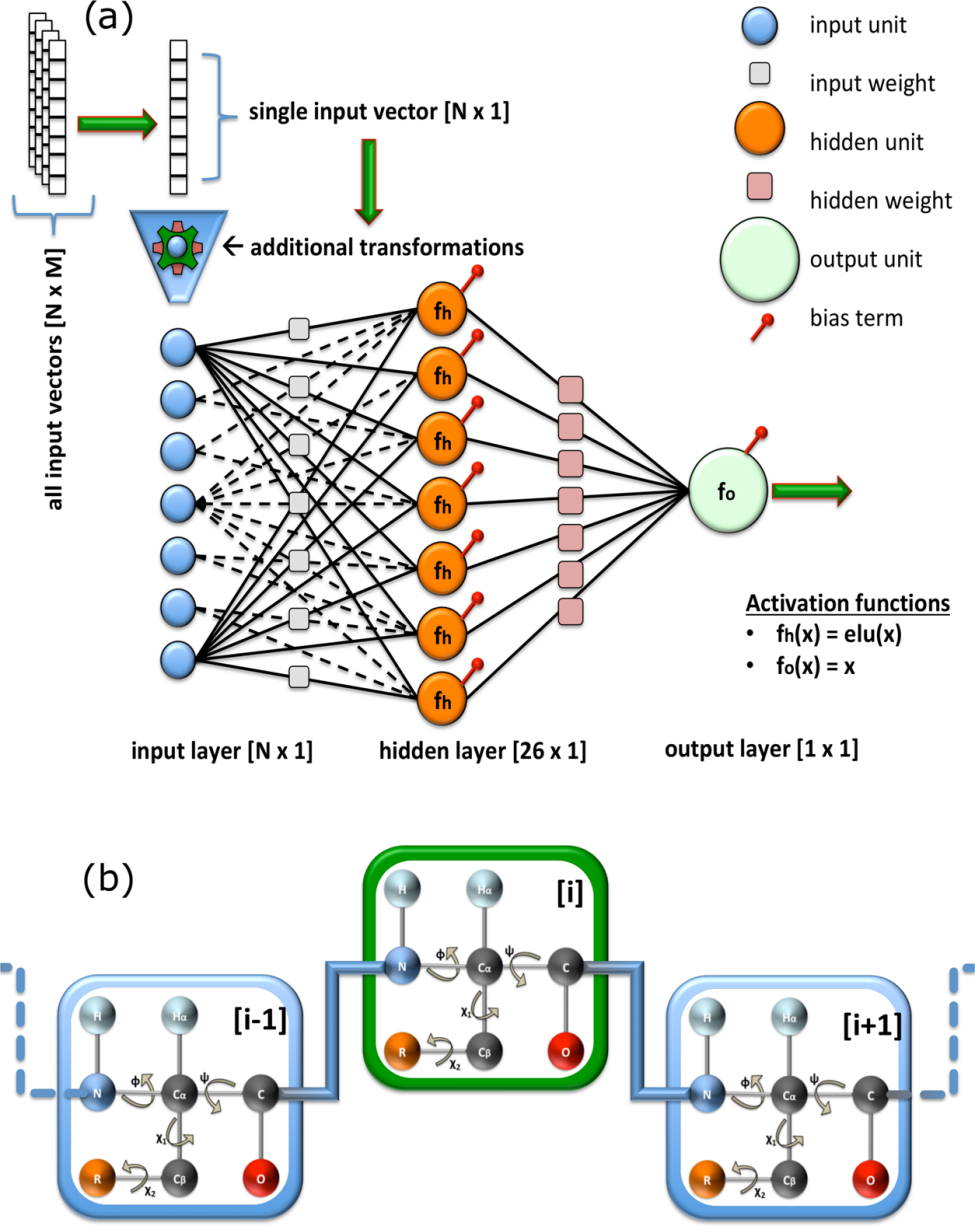
(a) Schematic representation of the proposed ANN, where *f*_h_ is the activation function of the hidden units, *f*_o_ is the activation function of the output unit, *N* denotes the number of training features, and *M* is the total number of training samples. The function elu(*x*) evaluates to α(exp(*x*) −1) when *x* < 0, where α > 0 is a constant, and *x* when *x* ⩾ 0. Note that not all input weights (gray squares) are drawn in the network to avoid cluttering the figure. (b) Simplified illustration of a tripeptide chain. The residue in the middle, with index *i* (green square), is the one that is targeted during the prediction. The neighboring residues, with indices *i* – 1 and *i* + 1 (blue square), provide additional local information.

Our ANNs consisted of: (i) an input layer, which receives the input vectors; (ii) a single hidden layer, which identifies a mapping between the inputs and outputs; and (iii) an output layer that provides the final prediction (Figure 1a).

We tested the use of mono-, tri-, penta-, and heptapeptide-based structural parameters to generate the input vectors for the ANN training. The best compromise between the accuracy and complexity of the calculation was found for tripeptides, in line with the previous development of SPARTA+.^8^ Thus, for a given residue *i* of the protein, the parameters employed in the input layer included: (i) the amino acid type and (ii) the torsion angles ϕ, ψ, χ_1_, and χ_2_ of the residues *i*, *i* – 1, and *i* + 1 (Figure 1b).

The protein sequence was processed in the ANN via BLOSUM62 to align the input sequence to the database. No significant alterations in the accuracy of predictions were observed when using other BLOSUM matrices (e.g., 45, 80, 90). In our parameterization, we used 22 amino acid types, as we distinguished between oxidized and reduced cysteine residues (named CYO and CYR, respectively), as well as cis and trans proline residues (named PRC and PRT, respectively).

The torsion angles ϕ, ψ, χ_1_, and χ_2_ were treated using their sine and cosine values (e.g., [sin ϕ, cos ϕ]). If an angle was missing, either because it could not be defined or because of numerical issues in its calculation, we defined the [sin ϕ, cos ϕ] couple as [0, 0]. This setting enabled us to avoid ambiguities during training and inform the ANN that the particular angle was missing since there is no angle that has its sine and cosine values simultaneously equal to zero.

Collectively, the parameters of each residue of the protein amounted to 30 numerical values:

- 22 from the BLOSUM62, and
- 8 from the cosine and sine values of each torsion angle.

As a result, the input to the ANN associated with a tripeptide was composed of 90 values (i.e., a column vector with dimensions [90 × 1], *N* = 90 in Figure 1a).

The hidden layer of the ANN consisted of 26 nonlinear units (or neurons). This is the layer that constructs the mapping between the input signal and the output target values during the training process. The nonlinear activation functions present in the hidden layer are essential for capturing the relatively complex relationships of the problem at hand. We chose the exponential linear unit function,^28^ defined as
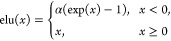
1where α > 0. The output layer consisted of a single unit with a linear activation function
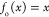
2This choice is typical for an output activation function in regression problems when the output of the model must be a continuous real value.

#### 2. Training

Training was carried out independently for each backbone atom type, leading to six independent ANNs. As the six atoms considered here were not always simultaneously present in BMRB entries, the sizes of the six training sets varied from 192,426 for C atoms to 253,929 for Cα atoms (see Table S1 in the Supporting Information). The N- and C-termini of each amino acid chain were treated using a zero-padding technique. With this approach, a fictitious “**0**” residue was added before the N-terminal and after the C-terminal residues, represented by 30 **zero** values in the input vectors. Overall, from the 2987 PDB files (Table S3 in the Supporting Information), 285,239 tripeptides were generated (including the zero-padded terminal entries).

Before fitting the ANN with this training set, we applied data scaling to obtain input values distributed in the same range. This data preprocessing step is generally required by machine learning algorithms, such as neural networks, to avoid artifacts during training. In particular, since the torsion angles were encoded by their sine/cosine values, they range by definition from −1 to 1. To normalize the values from the BLOSUM62 matrices into the same range, we scaled each feature by its maximum absolute value. This estimator is known as MaxAbsScaler^1^. Since this scaling does not center or shift the data, it did not alter any zero entries in the input vectors that were used to signal the absence of torsion angles.

We also applied an early termination criterion in our model training to avoid overfitting. Data overfitting is a common problem resulting from the complex formulation and the number of trainable parameters in neural network models. In particular, an ANN can in principle model not only the *signal* from the training set examples but also the *noise*. One approach to avoid overfitting includes the application of regularization to the cost function, which will either set many weights to zero (e.g., in L1 regularization) or penalize weights with large magnitudes (e.g., in L2 regularization). In NapShift, instead of using a complex model composed of several hidden layers, or a large number of hidden units requiring L1 and/or L2 regularization, we employed a single hidden layer with a small number of hidden units (e.g., 26) and combined this architecture with an early stopping condition to terminate the training as soon as overfitting was detected. This criterion was imposed on a *validation set* (usually around 10% of the training data) and monitored the loss function. Once the validation loss remained unchanged for a predefined number of iterations, the calculation terminated and returned the best solution found.

The ANN output was set to target secondary CS (i.e., Δδ*X*), an approach that has been successful in SPARTA+. The secondary CS is defined as
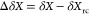
3where δ*X* is the experimentally observed CS; δ*X*_rc_ is the random coil CS; and *X* is one of the backbone atoms N, C, Cα, Cβ, H, or Hα. This substitution (eq 3) has the effect of a standardization that brings all target values to similar ranges. We used the CamCoil algorithm^29^ to calculate the random coil CS (δ*X*_rc_).

#### 3. Output function

The complete output function of the ANN model^2^ is given by
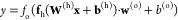
4where *y*_[1×1]_ is the scalar output of the network (i.e., Δδ*X*), *f*_o[1×1]_ is the scalar activation function of the output unit, **f**_*h*[26×1]_ is the activation function of the hidden layer units, **x**_[90×1]_ is the input vector, **W**_[26 × 90]_^(h)^ are the weights of the hidden layer units, **b**_[26×1]_^(h)^ is the bias vector of the hidden layer units, **w**_[26×1]_^(o)^ are the weights of the output layer, and *b*_[1×1]_^(o)^ is a scalar bias term of the output unit. Because *f*_o_ was chosen as a linear function (see eq 2), eq 4 simplifies to
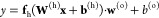
5

The hidden layer activation function, **f**_h_, was chosen as the exponential linear unit (see eq 1). In our formulation, we set α = 1, giving
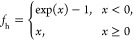
6

Including the bias term vector **b**^(h)^ in the weights matrix **W**^(h)^, we obtain: W_*_^(h)^ = [**W**^(h)^; **b**^(h)^]_[26×91]_ with one extra column, which allows us to further simplify eq 5 as
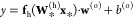
7where the input column vector x_*_ = [**x**; 1]_[91×1]_ has been augmented to include an additional 1 at the end.

### C. Hybrid Potential

The advantage of the definition of the ANNs of NapShift is that the energy penalty can be differentiated with respect to the Cartesian coordinates to obtain an “AI-based” CS restraint for structural refinement. To compute the derivatives of the final output function of eq 7 with respect to the torsion angles, the derivatives of the activation function eq 6 with respect to the input *x* were required:
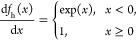
8

The derivative of eq 7 with respect to an angle θ (representing ϕ, ψ, χ_1_, or χ_2_) was obtained using the chain rule
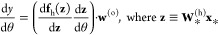
9d**f**_h_(**z**)/d**z** is obtained using eq 8 according to the input values of **z**, and d**z**/dθ contains mostly zeros, except where we have terms involving sin θ and cos θ.

For each dihedral angle included in the NapShift parameterization (θ = ϕ, ψ, χ_1_, or χ_2_), we calculated the force applied on each of the four atoms defining the angle. We obtained this force using the chain rule and the Cartesian derivatives of the sine and cosine of θ with respect to the atomic coordinates **r**
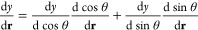
10

The forces applied arise from a harmonic potential for the back-calculated CS value, given by
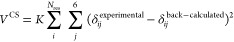
11where *V*^CS^ is the hybrid restraint potential energy, δ_*ij*_^experimental^ is the experimental secondary chemical shift, δ_*ij*_^back-calculated^ is the NapShift-predicted chemical shift (i.e., the output *y*), *i* and *j* represent the residue number and backbone atom type, respectively, and *K* > 0 is the weight of the restraint with respect to the empirical force field. For every CS input, forces were calculated and applied individually for all atoms involved in the dihedral angle of the tripeptide employed in the NapShift calculation: ϕ, ψ, χ_1_, and χ_2_.

The total potential energy, *V*^Total^, used in our calculations is therefore given by
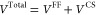
12where *V*^FF^ is the potential energy of the underlying biomolecular force field.

We have implemented the NapShift hybrid restraint potential in the GMIN^30^ and OPTIM^31^ programs for use with the Amber^32−34^ and CHARMM^35^ molecular mechanics force fields. The NapShift potential is also implemented in the GROMACS^25^ software package for MD simulations.

### D. Basin-Hopping Global Optimization

Basin-hopping (BH) global optimization^16−18^ was used to explore the impact of our hybrid potential on protein structure prediction simulations using the GMIN^30^ program. In each BH step, we propose a random perturbation of coordinates, locally minimize the resulting structure, and accept or reject the result based on a Metropolis criterion. For each production run, *k*_B_*T* in the Metropolis test was set to 1.0 kcal mol^–1^, and 100,000 BH steps were performed. Random perturbations for protein structures were proposed using the group rotation scheme,^36,37^ where backbone and side-chain dihedral angles are randomly selected and perturbed, which is more efficient than performing moves in Cartesian space. To ensure an appropriate combination of local and global moves during BH, dihedral angles were selected to allow for side-chain rotations, global backbone dihedral rotations, and constrained backbone dihedral rotations, where two Cα atoms are fixed and only the atoms in between the Cα atoms are allowed to rotate. Local minima were converged to a root-mean-square (RMS) gradient of 10^–3^ kcal mol^–1^ Å^–1^ after each BH step, then converged further to 10^–6^ kcal mol^–1^ Å^–1^ during refinement of the 50 lowest-energy structures. Optimization was performed using a modified version of the limited-memory Broyden–Fletcher–Goldfarb–Shanno algorithm.^38,39^

For our BH runs, we used a mixing parameter, α, rather than the weight *K*, and normalized by the number of residues, *N*_res_, to give the following restraint energy and total potential energy
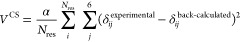
13
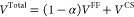
14This formulation allowed us to determine the effect of the hybrid potential as a function of the relative weight between the force field energy and the restraint energy over a number of different systems.

A tolerance parameter ϵ was also introduced to account for systematic error in the ANN prediction when evaluating the energy penalty
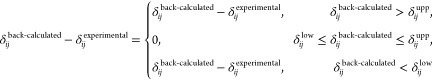
15where
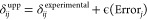
16
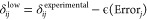
17This parameter creates a flat-bottom restraint function, where all predictions within a certain tolerance of the experimental value will lie at the bottom of the potential. This softens the contribution of the hybrid restraint forces in the case that the NapShift-predicted CS slightly deviates from the reference values. Error_*j*_ for each backbone atom type *j* corresponds to the root-mean-square deviation (RMSD) error of the NapShift-predicted chemical shifts for the given atom type over the testing dataset. For each of our BH runs, we used a tolerance of ϵ = 0.25.

### E. Discrete Path Sampling

We employed the discrete path sampling framework^19−21^ to generate kinetic transition networks and analyze the energy landscapes of each benchmark protein system at varying values of α, the hybrid mixing parameter. Kinetic transition networks were set up by first finding a selected discrete path between an initial unfolded minimum and the global minimum, identified by BH global optimization. Initial discrete paths were identified using the missing connection algorithm^40^ to identify gaps in a pathway between the two minima, then using the doubly nudged^41^ elastic band (DNEB)^42,43^ approach to identify candidates for intervening transition states, which were refined using the hybrid eigenvector-following scheme.^44−47^ The minima connected to each transition state were identified by following the two steepest-descent paths. All new minima and transition states found were then included in the missing connection analysis at the start of the next cycle. This procedure was continued until a connected path was found between the two initial minima. The initial path was then expanded using various procedures that reduce the number of transition states in a path, eliminate high energy barriers, and remove artificial frustration from undersampling, until the network converged.^40,48^ The energy landscapes that resulted from these minima and transition states and their connected paths were then visualized using disconnectivity graphs.^49,50^ This discrete path sampling scheme is implemented in the OPTIM^31^ and PATHSAMPLE^51^ programs.

### F. Restrained Molecular Dynamics Simulations

To study the dynamics of proteins in or near their free energy minimum, we implemented CS restraints in the GROMACS package for MD simulations,^25^ which allows for the use of a variety of force fields for proteins, water molecules, and lipids, as well as a large number of integration methods, such as replica averaging^52^ and meta-dynamics.^53^ The restraints were imposed by adding an experimentally driven energy term to the standard force field (eq 12), where the experimental term was modeled as a harmonic potential based on the calculated CS value (eq 11). The harmonic restraints were again implemented as flat-bottom potentials (eq 15), where no restraining force was applied within the experimental error limits of the measured values. The weight *K* of the restraint energy with respect to the empirical force field was increased from zero up to a maximum value during the initial equilibration simulation, and subsequently maintained constant during final sampling. In this study, the restraints were imposed directly on single copies (replicas) of the system, although the implemented MD restraints can also be used in ensemble-averaged MD simulations, which are better suited for heterogeneous systems.

MD simulations were run using the CHARMM36 force field^54^ and TIP3P explicit water model^55^ in the NPT ensemble by weak coupling of the pressure and temperature with external baths. Temperature coupling was performed with the V-rescale method^56^ using a coupling constant of 0.1 ps and a reference temperature of 300 K. The pressure was kept constant using the Berendsen method,^57^ with a compressibility value of 4.5 × 10^–5^ bar^–1^. Electrostatic interactions were treated using the particle mesh Ewald method.^58^ The integration step for the simulations was 2 fs, and the restraints were applied at each integration step. All MD simulations were carried out using periodic boundary conditions and adopting LINCS as a constraint algorithm.^59^

## III Results

### A. Predicting CS from Structure Using NapShift

Six NapShift ANNs were defined for the atoms N, C, Cα, Cβ, H, and Hα, using tripeptides derived from 2987 protein structures for which CS assignment was available. The performance of NapShift was tested using a set of 250 proteins that were not used in the training/validation phase (see Table S4 in the Supporting Information). This set was randomly selected from an initial database of 3237 PDB files, each associated with a corresponding BMRB entry. The only requirement for the 250 entries was that they were deposited after 2010, which provided an unbiased comparison with other programs such as SPARTA+ (published in 2010).

**Figure 2:**
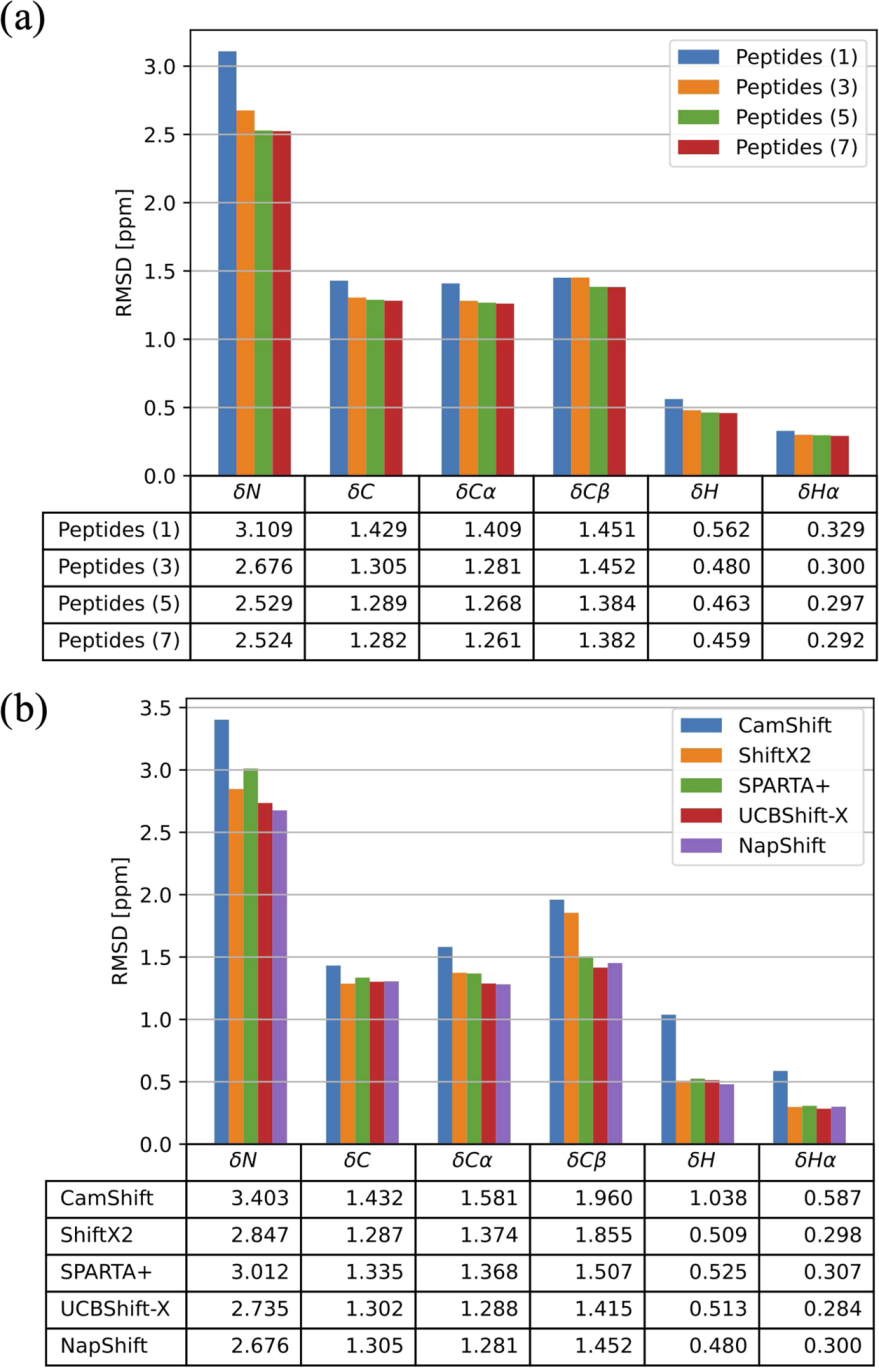
(a) Comparison of the RMSD in ppm between the NapShift-predicted CS and the experimental CS for varying input peptide lengths (1, 3, 5, and 7) to the NapShift ANN. (b) Comparison of the RMSD between predicted and experimental CS for five different prediction methods. NapShift outperforms most of the prediction methods, for all backbone atoms, on the same set of 250 test PDB structures. In the case of CamShift, a 30 ppm threshold on the CS predictions was applied to avoid systematic errors that would distort the RMSD values.

The RMSD of CS values predicted for the 250 testing set showed an improved accuracy upon increasing the length of the input peptide *n* from 1 to 7 (Figure 2a). To balance the accuracy and complexity of the computations, an architecture based on tripeptides was chosen.

To test the overall accuracy of NapShift, we compared it with four other known methods for CS prediction: (1) CamShift,^7^ (2) ShiftX2,^60^ (3) SPARTA+, and (4) UCBShift-X.^61^ Compared to CamShift, a CS prediction method that has also been implemented in structure refinement protocols, NapShift provides significant increases in prediction accuracy for all target atoms. To verify the statistical significance of the results, for each individual atom predicted, we performed a **t-test** between our method and all of the other methods (Table S2 in the Supporting Information).

Significant improvement was also found when comparing NapShift to ShiftX2 for the atoms N, Cα, Cβ, and H, while our predictions were comparable for C and Hα. In comparison with SPARTA+ and UCBShift-X, NapShift shows a smaller improvement in prediction accuracy. However, unlike SPARTA+ and UCBShift-X, the NapShift ANN formulation can be conveniently differentiated with respect to Cartesian coordinates, thereby enabling us to implement this method as a restraint for structure refinement. Taken together, our results indicate that the accuracy of NapShift in predicting CS from structure makes it a useful method for the development of hybrid restraint potentials for biomolecular simulation.

### B. Neural Network Training Cross-Validation

As an additional validation of the model architecture, we also used BH global optimization to train a set of NapShift ANNs. To apply BH global optimization to neural network training, we considered the input weights **W** as the “coordinates” of the system.^62^ We then optimized these weights by globally minimizing a mean-squared-error loss function with an L2 regularization term
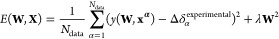
18where the output function *y*(**W**, **x**^***α***^) used the same input data, ANN architecture, and activation function as described above. The inputs were normalized by the absolute mean of each input column, and the L2 regularization value, used to prevent overfitting, was set to λ = 10^–5^.

Six different neural networks were again trained to generate secondary chemical shift predictions for the N, C, Cα, Cβ, H, and Hα atoms of a protein backbone. For each ANN, 500 BH steps were taken to optimize the network weights. The RMS gradient convergence criterion for initial quenches was chosen as 10^–3^ (unitless), then tightened to 10^–6^ for tight final quenches of the lowest minima. Using this independent training method, the NapShift architecture achieved similar success for the testing set of 250 proteins (Table I). The neural network weights obtained via BH global optimization achieved slightly more accurate predictions, so these weights were used for energy landscape analyses of the hybrid restraint potential.

**Table I:** RMSD of CS Values Predicted by the Original NapShift Model Trained Using Standard Python Machine Learning Tools and the NapShift Model Trained Using BH Global Optimization

atom type	N	C	Cα	Cβ	H	Hα
original RMSD	2.676	1.305	1.281	1.452	0.480	0.300
basin-hopping RMSD	2.645	1.300	1.269	1.451	0.472	0.296

### C. Energy Landscape Analysis

#### 1. Tryptophan Zipper 1

The effect of varying mixing parameters on the hybrid energy landscape for tryptophan zipper 1 has previously been examined^63^ using the CamShift method^7^ for calculating NMR chemical shifts alongside the CHARMM22 force field.^64^ Here, we performed a similar analysis using the NapShift neural network combined with the Amber ff99SB-ILDN force field.^65^ Reference chemical shifts used to represent the target structure were calculated using NapShift for the unoptimized PDB structure. Mixing parameters of α = 0, 0.3, 0.5, and 0.7 were considered, and 10 independent BH global optimization simulations for each value of α were run for 100,000 steps, with each run starting from a different random extended structure of the tryptophan zipper 1 sequence. Larger values of α were not explored, as the contribution of the biomolecular force field becomes too small, resulting in unphysical structures.

The lowest-energy structure from each BH run was analyzed using the two structural order parameters identified in the previous study.^63^ The first order parameter, O1, represents the number of native backbone hydrogen bonds correctly formed by the structure. Hydrogen bonds were identified using the default geometrical definition in the Amber CPPTRAJ program.^66^ Four backbone hydrogen bonds were identified in the experimental β-hairpin structure, giving O1 a maximum value of four. The second order parameter, O2, denotes the number of distances between centers of mass of neighboring pairs of tryptophan side chains that match the experimental structure to within ±0.5 Å for the two closest pairs and ±1.0 Å for the other pair. The maximum value of O2 is three, which would indicate the correct packing of all four tryptophan side chains. The lowest-energy structures were also evaluated by their RMSD compared to the experimental structure.

**Figure 3:**
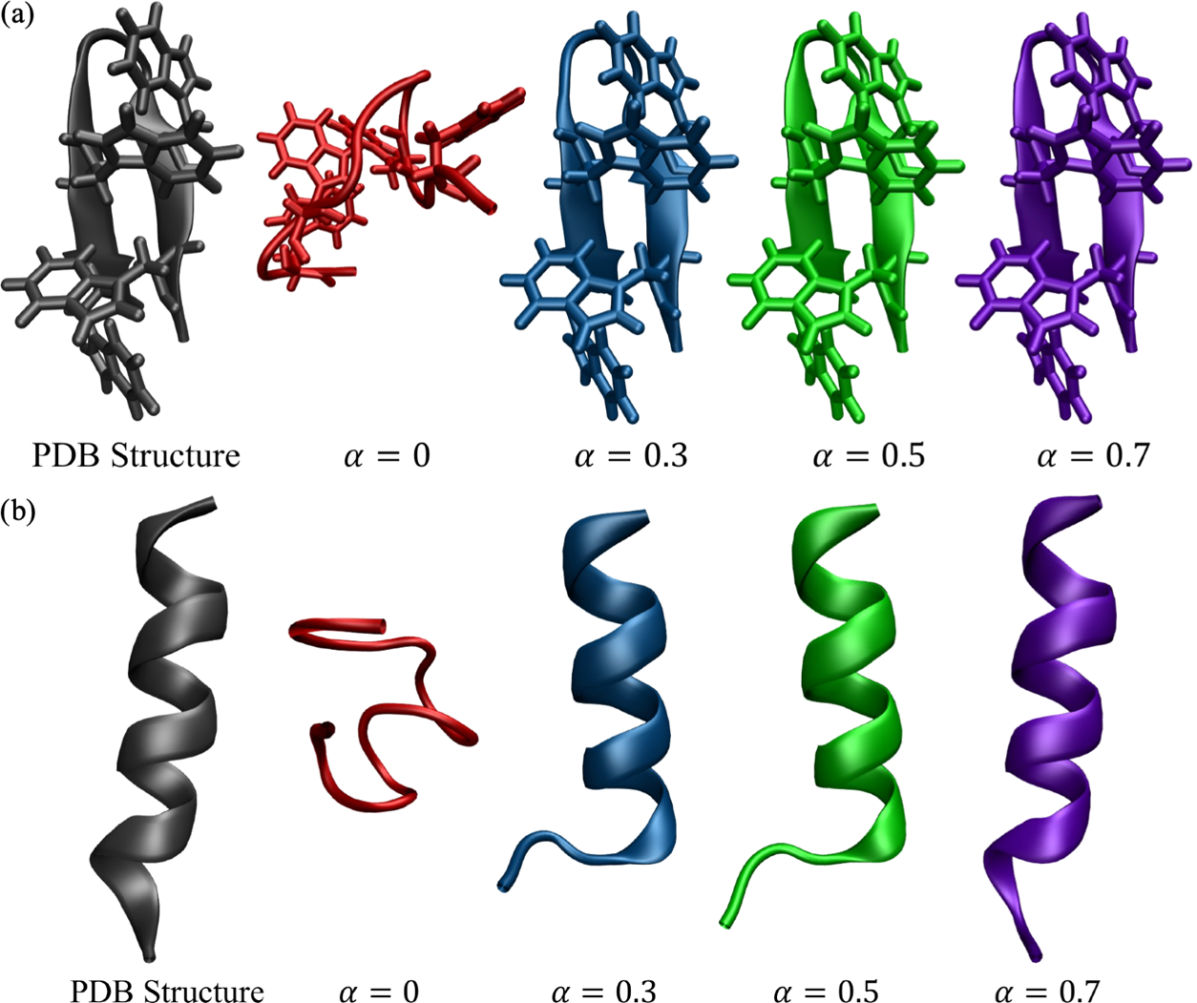
Experimental structure of (a) tryptophan zipper 1 (PDB code 1LE0(14)) and (b) a designed hydrophilic amphipathic basic helical peptide (PDB code 1DN3(15)), followed by structures located by BH global optimization at different values of the hybrid potential mixing parameter α. The structure shown for each α value is the minimum-energy structure obtained out of all 10 BH runs.

Energies, order parameters, and RMSD values for each of the lowest-energy structures from the BH runs are given in Table II. For α = 0 (i.e., no experimental restraints), none of the 10 BH runs were able to find a minimum-energy structure that exhibited any of the native hydrogen bonds of the experimental structure. The 10 structures also significantly deviated in their final energies, showing that the independent runs did not all produce the same lowest minimum, and thus the unrestrained energy landscape is a challenging case for *ab initio* global optimization. The overall lowest-energy structure from the unrestrained BH runs (Figure 3a) does not come close to forming the expected β-hairpin structure.

**Table II:** Analysis of the Lowest-Energy Structures from Each of the 10 BH Runs Performed on Tryptophan Zipper 1 for Varying Values of α, the Hybrid Potential Mixing Parameter^a^

BH Run	1	2	3	4	5	6	7	8	9	10
α = 0										
*V*^Total^	–511.09	–512.38	–511.37	–510.94	–512.84	–511.69	–508.41	–514.76	–512.43	–509.54
O1	0	0	0	0	0	0	0	0	0	0
O2	0	0	0	0	1	0	0	0	2	1
RMSD	4.748	4.368	5.548	3.896	4.373	3.633	4.790	4.513	3.870	5.803
α = 0.3										
*V*^Total^	–354.48	–358.61	–353.51	–355.23	–359.32	–359.25	–355.50	–359.56	–359.82	–359.17
*V*^CS^	1.331	0.133	1.856	2.419	0.096	0.295	2.819	0.097	0.091	0.044
O1	0	4	0	0	4	4	0	4	4	4
O2	0	3	0	1	3	3	1	3	3	3
RMSD	1.856	0.286	1.692	2.902	0.384	0.350	3.104	0.387	0.296	0.297
α = 0.5										
*V*^Total^	–255.78	–256.51	–255.76	–255.40	–255.58	–255.86	–256.75	–256.35	–256.31	–255.34
*V*^CS^	0.170	0.059	0.162	0.654	0.103	0.069	0.136	0.572	0.231	0.478
O1	4	4	4	4	4	4	4	4	4	4
O2	3	3	3	1	2	3	3	2	3	0
RMSD	0.472	0.296	0.357	0.622	0.331	0.279	0.383	0.396	0.369	0.593
α = 0.7										
*V*^Total^	–152.77	–153.23	–153.02	–153.64	–153.51	–153.77	–152.81	–153.39	–152.76	–152.80
*V*^CS^	0.302	0.101	0.656	0.138	0.150	0.089	0.142	0.143	0.297	0.363
O1	4	4	4	4	4	4	4	4	4	4
O2	2	3	2	3	3	3	3	3	2	1
RMSD	0.454	0.387	0.361	0.367	0.275	0.346	0.358	0.38	0.458	0.482

a *V*^Total^ is the total potential energy, *V*^CS^ is the chemical shift restraint energy, O1 is an order parameter denoting the number of native backbone hydrogen bonds found in the structure (maximum four), and O2 is an order parameter denoting the number of distances between centers of mass of neighboring pairs of TRP side chains that match the PDB structure to within ±0.5 Å for the two closest pairs and ±1.0 Å for the other pair (maximum three). The RMSD is calculated between the experimental structure (pdb code 1LE0(14)) and the BH-predicted structure.

To determine whether a native-like lowest-energy structure could be found using the unrestrained potential, we started a BH global optimization run using the experimental tryptophan zipper structure as a starting conformation, rather than an extended structure. Indeed, the lowest-energy structure identified by this BH run displayed the correct hydrogen-bonding pattern. This result suggests there do exist low-lying minima on the unrestrained energy landscape that correspond to native-like structures, but these conformations are difficult to find within 100,000 BH steps. This situation is likely a result of the high energy barriers involved in rotating all four tryptophan side chains to the same side of the β-hairpin, and competing low-energy states with alternative structures for the unmodified potential.

Turning on the NapShift hybrid restraint potential immediately improved the performance of BH global optimization. For α = 0.3, six out of the 10 BH runs were able to find a lowest-energy minimum that reproduced all four of the native hydrogen bonds formed by the experimental structure. When the mixing parameter α was raised to 0.5, all 10 of the BH runs located a lowest-energy structure that reproduced the correct native hydrogen-bonding pattern. Six out of the 10 structures also exhibited the correct side-chain packing of all four tryptophan residues. Setting α = 0.7 gave similar results, with all 10 BH runs reproducing the native hydrogen-bonding pattern and 6 out of the 10 structures showing the correct tryptophan side-chain packing.

Incorporating the hybrid potential in our BH runs raised the energy of structures where the tryptophan side chains lay on opposite sides of the β-hairpin, as the incorrect backbone dihedrals incurred an energy penalty based on the CS restraints. This penalty reduced the energy barriers involved in forming the correct hydrogen-bonding pattern and side-chain packing, leading to more consistent identification of native-like structures.

**Figure 4:**
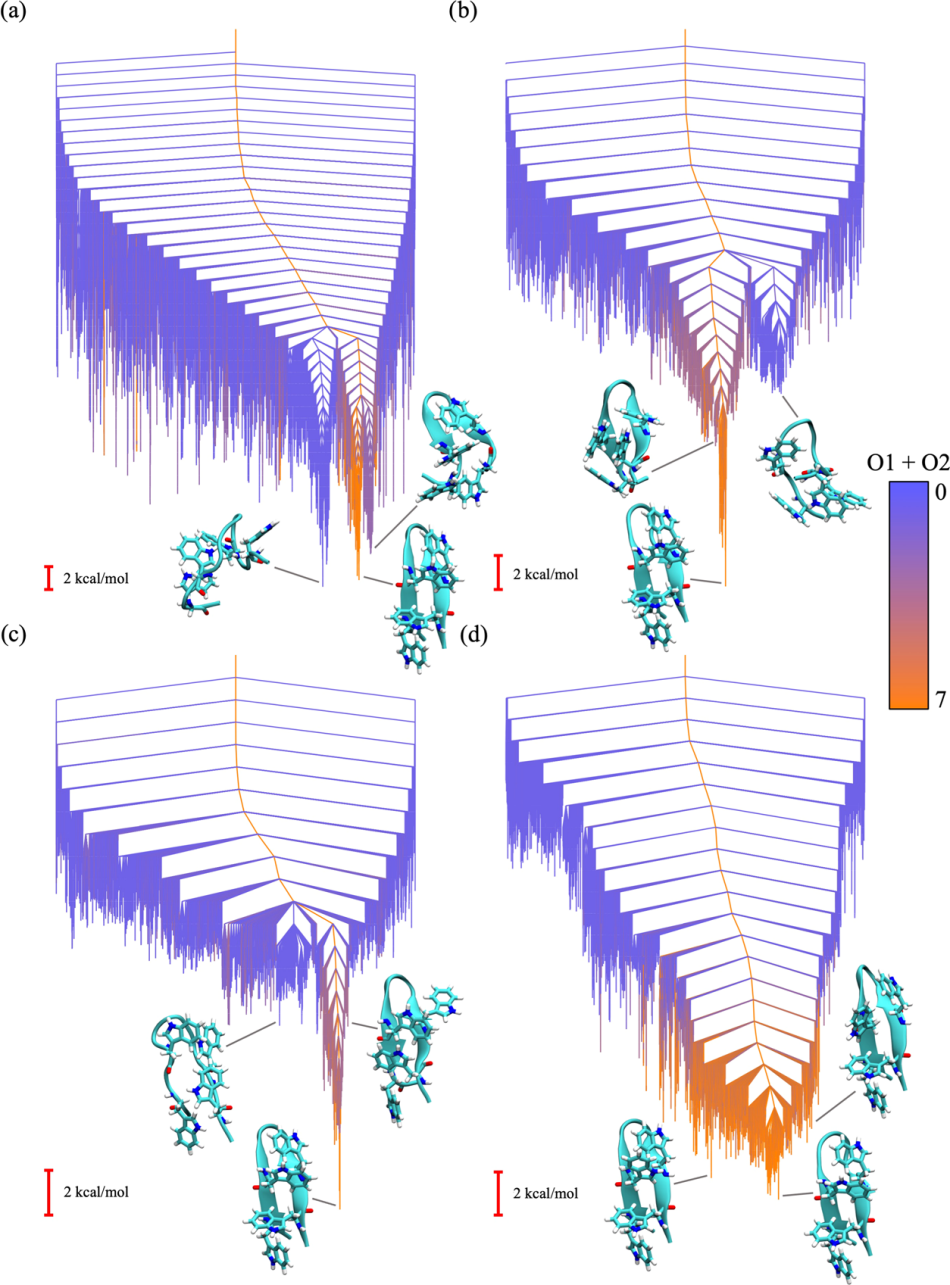
Disconnectivity graphs representing the energy landscape of tryptophan zipper 1 with hybrid potential mixing parameter values of (a) α = 0, (b) α = 0.3, (c) α = 0.5, and (d) α = 0.7. The coloring in the graph denotes the sum of the order parameters O1 and O2 calculated for each minimum in the energy landscape, where blue represents the minimum possible value (zero) and orange represents the maximum possible value (seven). The structures shown for each disconnectivity graph are selected minima from the corresponding energy landscape.

This result is consistent with the analysis of the global potential energy landscape using discrete path sampling^19−21^ as a function of the mixing parameter α. Disconnectivity graphs representing these landscapes are shown in Figure 4. The bottom of the unrestrained landscape (Figure 4a) has a prominent low-energy funnel containing non-native conformations (i.e., structures with the incorrect hydrogen-bonding pattern and tryptophan side-chain packing). A neighboring funnel that contained structures with more native-like characteristics is separated from this non-native funnel by a significant barrier. As we increase the α value to 0.3 and 0.5 (Figure 4b,c), the non-native funnel is penalized in energy by the hybrid restraint potential and the bottom of the landscape contains many more native-like conformations. For α = 0.7 (Figure 4d), the non-native funnel is essentially eliminated, as the majority of low-energy structures correspond to the native β-hairpin.

#### 2. Human Platelet Factor 4

One possible benefit of using a hybrid potential in the simulation of biomolecules is the ability to avoid the use of explicit or implicit solvent models. We anticipate that, in some cases, the use of experimental restraints may suffice to represent the effect of solvent on the system. To test this hypothesis, we examined a 15-residue model hydrophilic amphipathic basic helical peptide, designed by Montserret et al.^15^ The design of this peptide was based on the sequence of residues 56–70 of human platelet factor 4. The peptide was experimentally shown to be unstructured in water, but folded into an extended α-helix when placed in an SDS solvent. We performed 10 BH runs as above using the default generalized Born implicit solvent model to explore whether our hybrid restraint potential could replicate an SDS solvent without having to modify the existing solvent model parameters.

The lowest-energy structures from each BH run were analyzed using a single order parameter, O3, denoting the number of residues in the structure labeled as α-helical by STRIDE secondary structure assignment.^67^ The first and last residues of the peptide were not considered, giving O3 a maximum value of 13. The RMSD between the predicted and experimental structure was again calculated for each BH run.

Without any restraints, the lowest minima found by BH were disordered, which is consistent with the experiments performed in water.^15^ Only four out of the 10 BH runs found lowest-energy structures with any sort of helical secondary structure, and in each of these four cases fewer than half of the residues were identified as α-helical. When using the hybrid potential with α = 0.3, all 10 of the BH runs located structures in extended α-helical conformations, each with an O3 value of either 10 or 11. Setting α = 0.5 gave similar results, as each of the 10 BH runs produced a structure with an O3 value of 11. The main deviations from experiment in the structures produced by the BH runs with α = 0.3 and 0.5 were in the N-terminal ends of the proteins, where residues 2 and 3 were identified as random coil (i.e., lacked defined secondary structure). In the experimental structure, these residues contribute to the α-helix (Figure 3b).

A mixing parameter of α = 0.7 gave the best results, as each of the 10 BH-predicted structures had an O3 value of either 12 or 13, indicating a completely extended α-helix structure, as seen in experiment. The average RMSD to experiment of these structures was also much lower than for the structures produced by the α = 0.3 and 0.5 BH runs.

**Figure 5:**
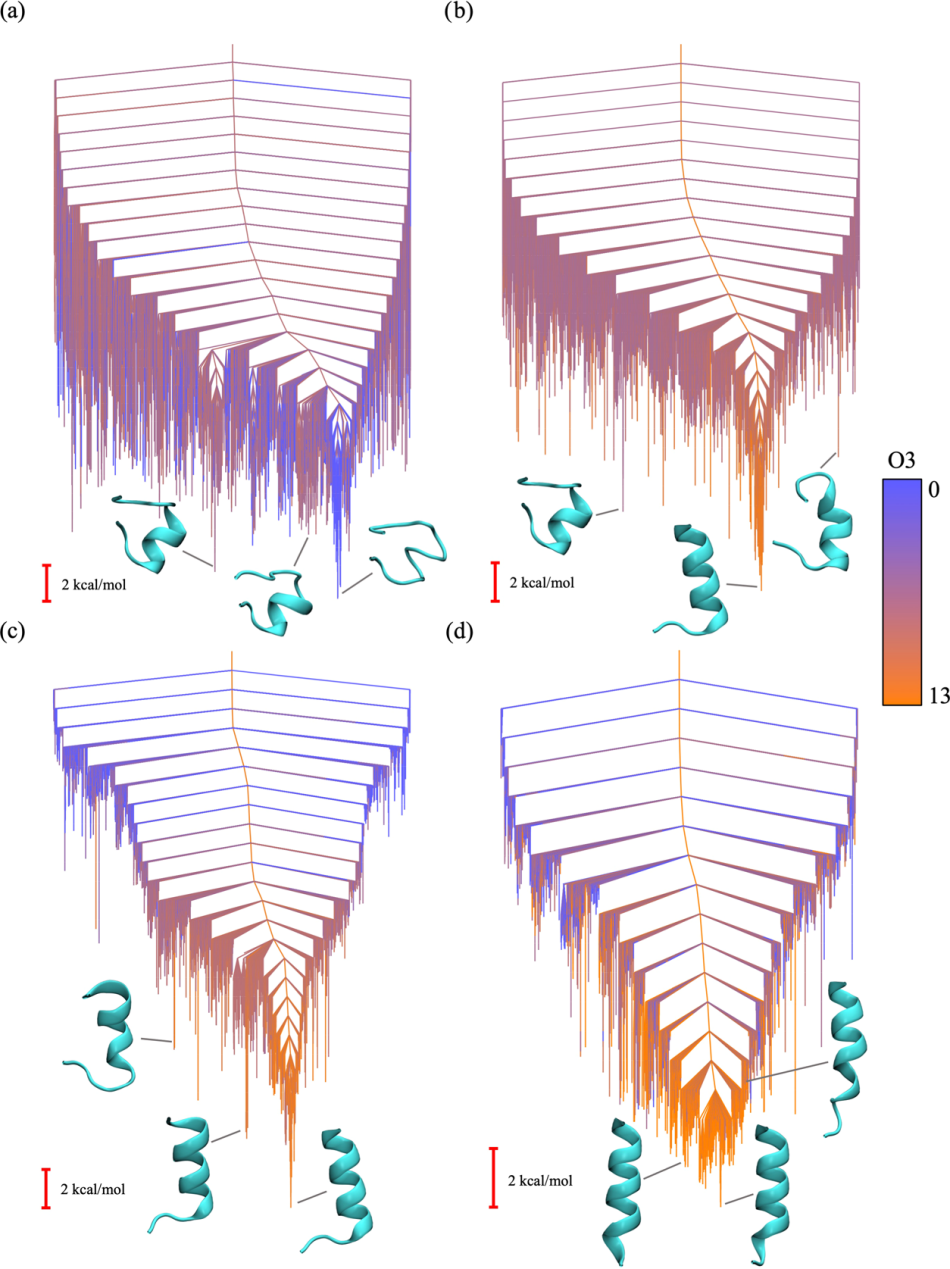
Disconnectivity graphs representing the energy landscape of 1DN3 with hybrid potential mixing parameter values of (a) α = 0, (b) α = 0.3, (c) α = 0.5, and (d) α = 0.7. The coloring employs the value of the order parameter O3 calculated for each minimum in the energy landscape, where blue represents the minimum possible value (zero) and orange represents the maximum possible value (13). The structures shown for each disconnectivity graph are selected minima from the corresponding energy landscape.

We again used the discrete path sampling method^19−21^ to analyze the underlying energy landscapes for each value of α, and the disconnectivity graphs are shown in Figure 5. The unrestrained potential energy landscape (Figure 5a) has a number of local minima with partial α-helices, but none with the extended α-helix conformation seen in the PDB structure. Furthermore, the lowest-energy funnel of the energy landscape corresponds to a conformation with no α-helical secondary structure. Incorporating the hybrid potential immediately introduced extended α-helical structures as low-energy minima into the resulting energy landscapes (Figure 5b–d). The landscape corresponding to a mixing parameter of α = 0.7 contains the most low-lying native α-helical conformations, as well as the lowest downhill barriers, which explains the improved performance of the BH runs for this value.

The success of global optimization in finding the extended α-helical conformation as the lowest-energy minimum shows that the hybrid restraint potential corrects for the errors introduced by the default Generalized Born implicit solvent instead of an explicit SDS solvent. This result suggests that experimental restraints could be used in place of more complicated solvent models to reduce computational cost and overall model complexity.

#### 3. Designed Peptide 5

DP5^22^ is an 18-residue designed peptide, experimentally shown to exist in two distinct folded conformations: an α-helix (PDB code 2DX3) and a β-hairpin (PDB code 2DX4). A previous computational study used snapshots from molecular dynamics simulations to suggest that DP5 has a multifunnel energy landscape, where competing α-helix and β-hairpin conformations make it difficult for global optimization methods to identify a native structure from the sampled minima.^68^ The authors of this previous study began MD simulations from each of the two experimental PDB structures, rather than attempting to sample the two competing conformations starting from an extended structure, as the relatively complex multifunnel energy landscape causes broken ergodicity problems.

We used the NapShift hybrid restraint potential to modify the energy landscape and guide BH global optimization runs toward each of the two competing DP5 experimental structures. Separate sets of reference CS used to represent the α-helix and β-hairpin conformations were calculated from the PDB structures following local minimization to an RMS gradient of 10^–3^ kcal mol^–1^ Å^–1^, which eliminated bad clashes in the original experimental structures. Three independent BH global optimization runs were started from an extended DP5 conformation: one without any experimental restraints, one with CS restraints based on the helical conformation, and one with CS restraints based on the hairpin conformation. For the restrained BH runs, the mixing parameter was set to α = 0.7, as this value gave the best results for the previous two benchmark systems. For each run, 100,000 BH steps were performed (Table III).

**Table III:** Analysis of the Lowest-Energy Structures from Each of the 10 BH Runs Performed on the Designed Helical Peptide for Varying Values of α, the Hybrid Potential Mixing Parameter^a^

BH Run	1	2	3	4	5	6	7	8	9	10
α = 0										
*V*^Total^	–511.85	–510.81	–514.28	–512.98	–513.82	–513.35	–512.76	–514.86	–510.35	–513.25
O3	0	0	0	5	0	5	6	0	0	6
RMSD	6.98	4.821	6.185	6.229	6.174	5.191	5.812	5.968	7.875	5.626
α = 0.3										
*V*^Total^	–355.22	–355.41	–355.43	–355.31	–354.92	–355.19	–355.51	–355.52	–355.09	–355.49
*V*^CS^	1.510	1.850	1.605	1.275	1.609	1.640	1.617	1.537	1.510	1.586
O3	11	10	11	11	10	10	10	11	11	10
RMSD	2.657	2.829	2.751	2.330	2.779	2.889	2.892	2.709	2.634	2.842
α = 0.5										
*V*^Total^	–251.68	–252.00	–252.59	–252.52	–252.02	–252.09	–252.27	–252.62	–252.49	–252.43
*V*^CS^	2.419	1.994	1.956	1.978	1.981	2.386	1.994	1.994	2.001	2.347
O3	11	11	11	11	11	11	11	11	11	11
RMSD	2.345	2.340	2.345	2.332	2.355	2.707	2.344	2.340	2.361	2.363
α = 0.7										
*V*^Total^	–149.99	–149.93	–150.11	–149.70	–149.71	–149.93	–149.98	–149.75	–149.71	–149.78
*V*^CS^	0.476	0.124	0.078	0.578	0.388	0.094	0.063	0.025	0.027	0.035
O3	13	12	12	13	13	12	12	12	12	12
RMSD	1.199	0.487	0.525	1.081	1.221	0.404	0.471	0.446	0.452	0.405

a *V*^Total^ is the total potential energy, *V*^CS^ is the chemical shift restraint energy, and O3 is an order parameter denoting the number of residues that are labeled as α-helical by the STRIDE secondary structure assignment software.^67^ The maximum value of O3 is 13, as the first and last residues of the structure are not considered. The RMSD is calculated between the experimental structure (PDB code 1DN3(15)) and the BH-predicted structure.

Without the hybrid potential, BH global optimization failed to find a minimum that displayed either of the competing forms of secondary structure. The lowest-energy structure identified by the BH run was highly disordered and did not resemble either the experimental helix or hairpin conformation. This result suggests that, when starting BH global optimization from an extended structure, it is difficult to locate the funnels corresponding to the helix or hairpin conformations within 100,000 BH steps due to the complexity of the underlying energy landscape.

**Figure 6:**
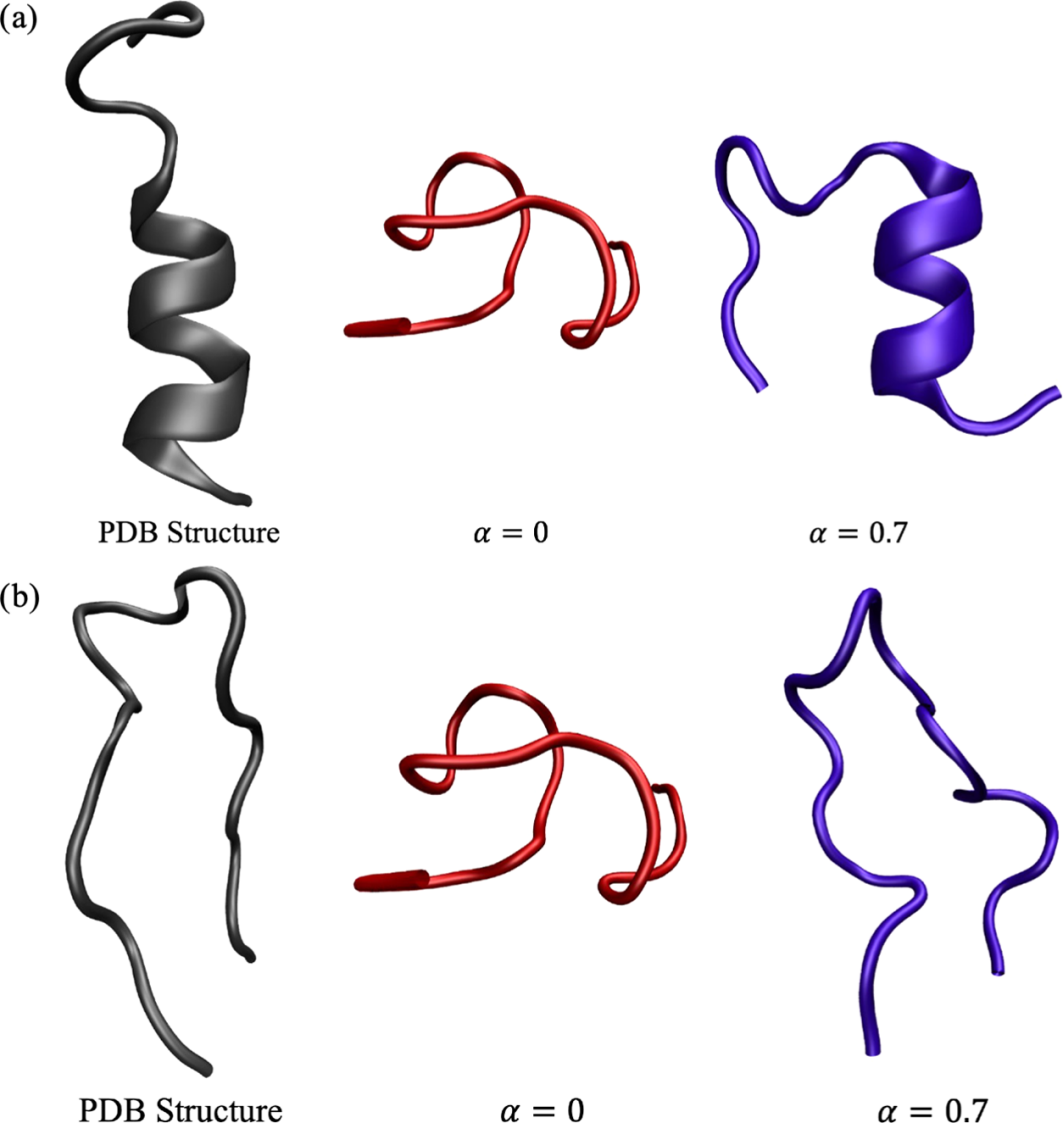
Experimental (a) α-helix and (b) β-hairpin structures of DP5, followed by the lowest-energy structures obtained from the unrestrained BH runs and the BH runs with CS restraints.

When incorporating NapShift CS restraints based on the helical conformation, BH global optimization was able to locate a lowest-energy structure that displayed α-helix secondary structure. Residues 3 through 10 were identified as α-helical by the STRIDE secondary structure assignment software,^67^ which is consistent with the experimental structure. Although the total RMSD of the global minimum to the experimental structure was slightly higher than for the structure obtained from the unrestrained BH run (Table IV), this deviation was purely due to differences in the highly flexible C-terminal region of the structure (Figure 6a). When considering only the α-helical residues, the RMSD of the restrained structure was 2.98 Å, while the RMSD of the unrestrained structure was 3.93 Å, showing significant improvement for structure prediction.

**Table IV:** Analysis of the Lowest-Energy Structures from Each of the BH Runs Performed on DP5: One with No CS Restraints, One with CS Restraints Based on the α-Helix Conformation, and One with CS Restraints Based on the β-Hairpin Conformation^a^

BH run	*V*^Total^	*V*^CS^	RMSD to helix	RMSD to hairpin
α = 0	*–*433.81	0.000	6.629	7.234
α = 0.7, helix	*–*127.91	1.063	7.060	5.049
α = 0.7, hairpin	*–*126.65	1.328	8.068	4.198

a *V*^Total^ is the total potential energy and *V*^CS^ is the chemical shift restraint energy. The RMSD was calculated between the experimental helix (PDB code 2DX3) and the BH-predicted structure, as well as the experimental hairpin (PDB code 2DX4) and the BH-predicted structure.

The restrained hairpin results were more ambiguous, as none of the residues in the experimental structure or the BH global minimum structures satisfied the STRIDE requirements for a β-bridge conformation. Previous studies have shown that NMR descriptions of a β-hairpin do not always satisfy the traditional geometric secondary structure criterion.^69,70^ Upon visualization of the resulting structures, it is clear to see that BH global optimization with the hybrid restraint potential located a hairpin-like global minimum structure, while the unrestrained lowest-energy structure (the same structure as analyzed previously in the α-helix case) does not take on a hairpin conformation (Figure 6b). The RMSD compared to the experiment of the lowest-energy structure from the restrained BH run was also much lower than that of the unrestrained BH run.

Thus, in both the α-helix and β-hairpin cases, hybrid restraints led to better sampling of the conformations associated with the corresponding competing form of secondary structure. Using a hybrid potential to guide the exploration of alternative morphologies in a disordered protein with a multifunnel energy landscape can therefore allow us to better analyze conformation-specific functions and dynamics.

#### 4. Ubiquitin

To test the NapShift hybrid restraint potential on a larger globular protein, we selected the well-studied system of ubiquitin, a 76 residue protein, which plays a key role in biomolecular signaling.^71^ Ubiquitination is a post-translational modification where one or more ubiquitin monomers attach to a substrate protein, which can determine the fate of the modified protein. For example, ubiquitination can be used to target a given protein for degradation^72^ or recruit proteins as binding partners for participation in certain biomolecular processes, such as DNA repair.^73^

Post-translational modifications of ubiquitin itself can also act as a biomolecular signal.^74^ One particular example involves phosphorylation of the serine residue at position 65, which is associated with the selective degradation of mitochondria.^75,76^ For this phosphorylation to occur, ubiquitin must take on a transient conformation, Ub-CR, in which the C-terminal tail is retracted and S65 is exposed.^23^ A recent study computational study explored the corresponding energy landscape underlying the transition between native ubiquitin (Ub) and Ub-CR, revealing a significant energy barrier between the two conformations.^24^

Using BH global optimization, we attempted to overcome this energy barrier and search from Ub-CR back to the native Ub conformation. Two BH runs were started from the Ub-CR conformation (PDB code 5XOI,^23^ with the serine at residue 67 mutated back to the native leucine using LEaP), which was initially locally minimized using only the Amber ff99SB-ILDN force field to eliminate any clashes. One BH run used only the original Amber ff99SB-ILDN force field, and the other included the NapShift hybrid restraint potential with an α value of 0.7. Experimental reference CS values for ubiquitin were obtained from BMRB entry 5387.^77^ The same BH parameters and group rotation scheme as described for the previous benchmark systems were used here, and 50,000 BH steps were considered.

Calculating the NapShift hybrid potential energy and gradient involves repeated multiplication of large matrices, which is required for neural network operations in the prediction operations. The cost of these calculations quickly becomes significant as system size increases. To use the hybrid potential with larger proteins, such as ubiquitin, we therefore implemented a GPU-accelerated version of NapShift. We achieved a 16-fold speedup over the original CPU algorithm using the GPU implementations of both NapShift and the Amber potential. This combination allowed us to complete 50,000 BH global optimization steps of ubiquitin in less than a week, a process that would take months to complete on one CPU.

To account for the larger system size, which resulted in force field energy and force values that were larger in magnitude, instead of normalizing the hybrid restraint energy and forces by the total number of residues, we normalized by the square root of the number of residues
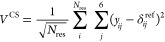
19This modification provided a balance between the force field and the hybrid restraint forces comparable to the values for the smaller benchmark systems considered above.

The similarity of a structure to either Ub or Ub-CR can be quantified by a simple order parameter, *q*, computed as the relative distance between the Cα atoms of residues 4 and 65 and residues 4 and 67. This choice measures the retraction of the C-terminal tail, which is the main structural difference between Ub and Ub-CR. The native conformation has a *q* value of 0.994, while Ub-CR has a *q* value of 1.745, as the retracted C-terminal tail leads to a greater distance between the phenylalanine at position 4 and the leucine at position 67. The structures obtained from BH were also evaluated in terms of their RMSD compared to both Ub and Ub-CR.

**Figure 7:**
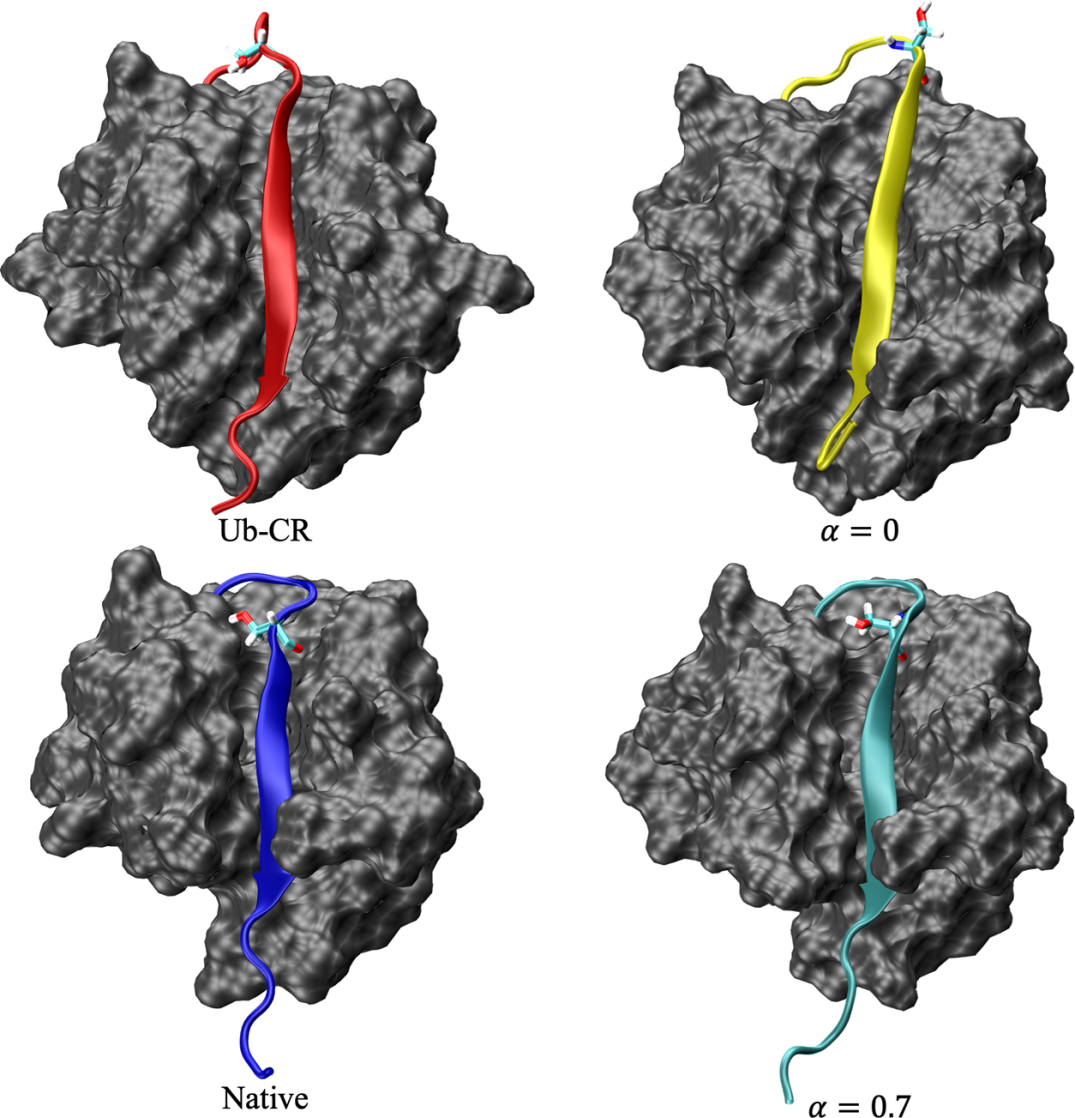
Native ubiquitin conformation (PDB code 1UBQ(78)), the Ub-CR conformation (PDB code 5XOI,^23^ with the serine at residue 67 mutated back to the native leucine using LEaP), and the lowest-energy conformations obtained from BH global optimization using only the original Amber ff99SB-ILDN force field (α = 0) and using the NapShift hybrid restraint potential (α = 0.7). The C-terminal tails are colored and residue S65 is explicitly identified in each structure. Without the hybrid potential, the structures sampled in BH remain closer to the Ub-CR conformation, shown by the retracted C-terminal tail (yellow) and exposed S65 residue. Running BH global optimization allows us to identify a native-like conformation, where the C-terminal tail (cyan) is extended and S65 is buried.

The lowest-energy structure from the unrestrained BH run remained in the Ub-CR conformation, where the C-terminal tail is retracted and residue S65 is accessible for phosphorylation (Figure 7). The value of *q* for this structure was 1.822, which is much closer to the *q* value of Ub-CR than Ub (Table V). This result suggests that it is difficult for BH to overcome the energy barrier associated with the Ub-CR to Ub transition within 50,000 steps.

**Table V:** Analysis of the Lowest-Energy Structures from Each of the BH Runs Performed on Ubiquitin^a^

BH run	*V*^Total^	*V*^CS^	*q*	RMSD to native	RMSD to Ub-CR
α = 0	–3447.82	0.000	1.822	3.008	1.705
α = 0.7	–1001.45	25.57	1.131	1.830	2.398

a Two BH runs were started from the wild-type Ub-CR conformation: one using only the original Amber force field and one including the NapShift hybrid potential with an α value of 0.7. *V*^Total^ is the total potential energy, *V*^CS^ is the CS restraint energy, and *q* is an order parameter defined as the ratio of the distances between the Cα atoms of residues 4 and 65 and residues 4 and 67. The RMSD was calculated between the BH-predicted structure and both the native ubiquitin conformation (PDB code 1UBQ(78)) and the Ub-CR conformation.

The hybrid potential BH run produced a lowest-energy structure much closer to the native fold, in which the C-terminal tail is extended and residue S65 is buried (Figure 7). The resulting structure has a *q* value of 1.131 and an RMSD of 1.83 Å to the native structure (Table V). Incorporating the NapShift hybrid potential facilitates the transition required to locate the native ubiquitin conformation. BH global optimization was therefore able to navigate the landscape for the hybrid potential and locate the global minimum within 50,000 steps.

### D. Restrained Molecular Dynamics Simulations

**Figure 8:**
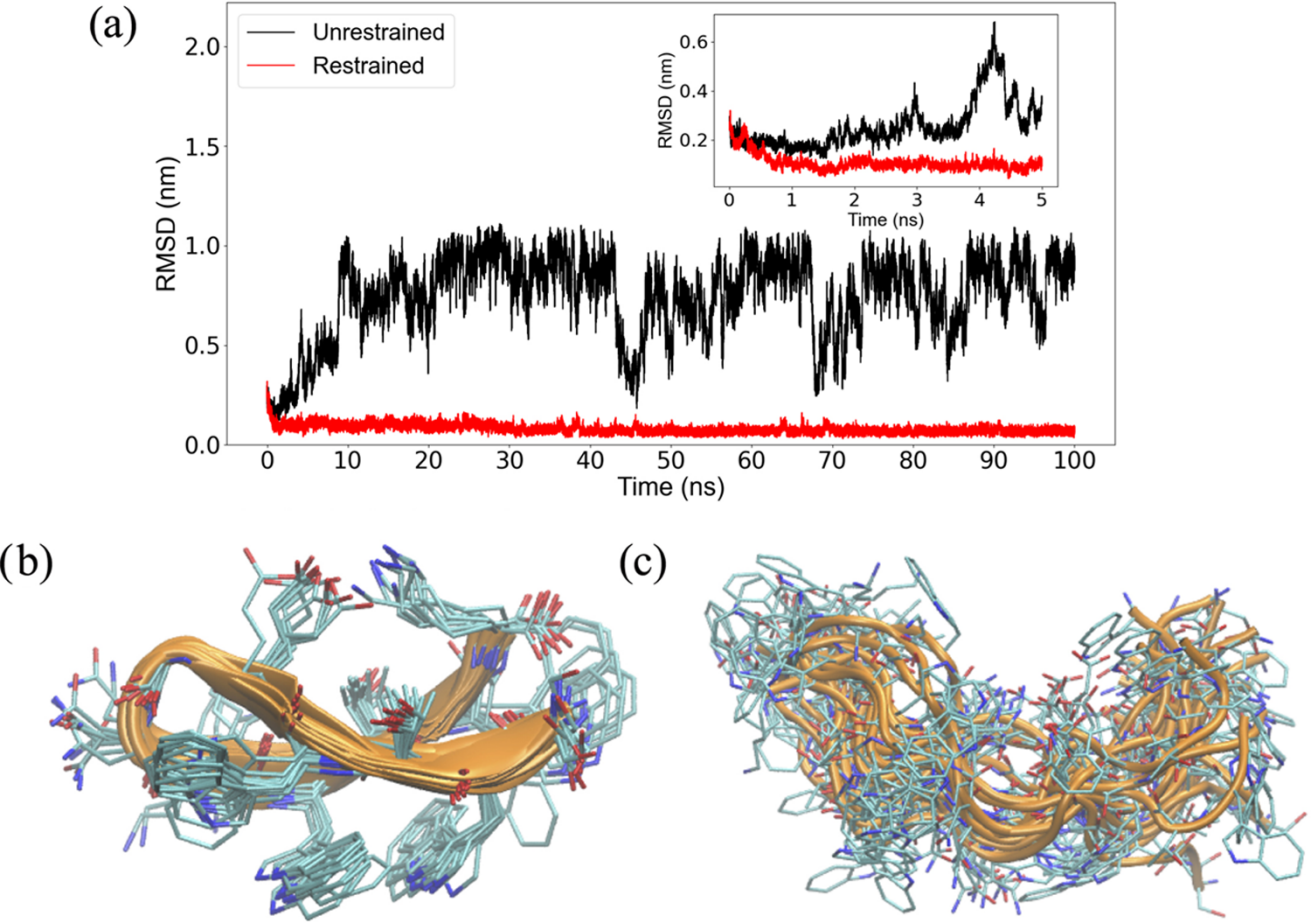
MD simulation results for tryptophan zipper 1. (a) RMSD to experiment over restrained and unrestrained 100 ns MD simulations starting from a distorted conformation of the β-hairpin. In the restrained simulation, the restraining force was linearly increased from 0 to a maximum value of 600 J mol^–1^ ppm^2^ over the first 12 ns of sampling. (b, c) Ensembles of conformations sampled in (b) unrestrained and (c) restrained simulations. The conformations were extracted from the last 60 ns of the MD trajectories.

In addition to our energy landscape analysis, we explored the ability of CS restraints to improve the accuracy of force fields for MD simulations. We again used tryptophan zipper 1 as a test case and ran simulations with the CHARMM36 force field^54^ and the TIP3P explicit water model.^55^ The tight packing of tryptophan side chains in the β-hairpin led to local stability on the MD timescale in both restrained and unrestrained MD simulations when starting from the native structure. Thus, to test the quality of the CS restraints, we started our simulations from a distorted conformation of the peptide, obtained from an initial high-temperature (500 K) MD simulation (2.7 Å RMSD from the experimental structure). MD simulations of 100 ns were run as restrained and unrestrained in the NPT ensemble at a pressure of 1 atm and a temperature of 300 K. Over the course of the unrestrained simulation, the structure lost its native packing and adopted misfolded conformations with RMSD values reaching 10 Å from the experimental structure (Figure 8a,b). In contrast, an MD simulation with CS restraints returned the β-hairpin into its native conformation (Figure 8a–c) to within RMSD values of about 1 Å.

**Figure 9:**
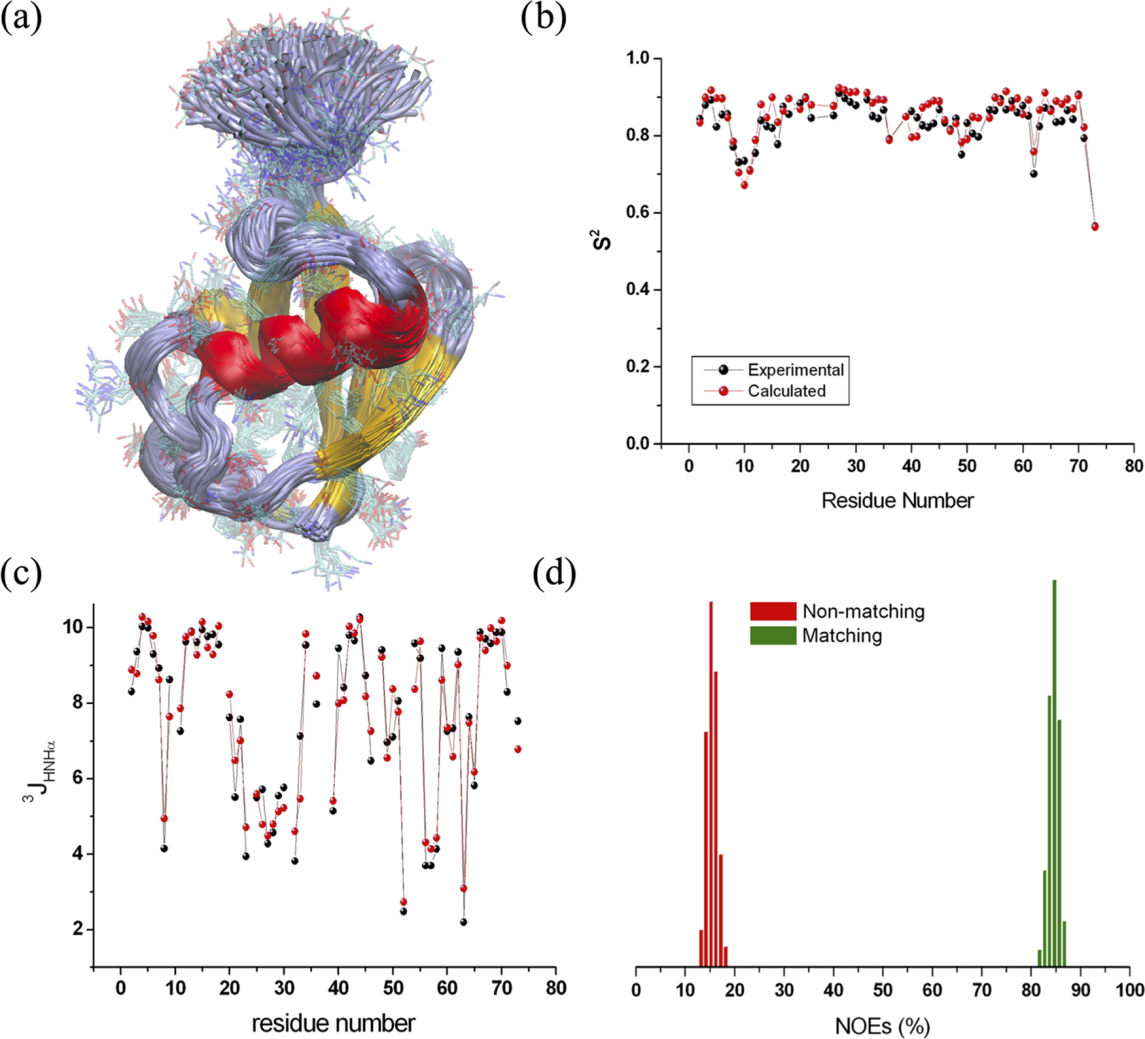
MD simulation results for ubiquitin. (a) Ensemble of structures sampled with restrained MD. (b) Agreement between experimental and back-calculated *S*^2^ values. (c) Agreement between experimental and back-calculated ^3^*J*-coupling values. (d) Distribution of back-calculated NOEs satisfying or violating the experimental values in the structures of the ensemble.

We used the same protocol for a restrained MD simulation of ubiquitin to sample its dynamics on the nanosecond timescale. Starting from an experimental structure (PDB code 2LJ5(79)), the restrained simulations generated a structural ensemble in excellent agreement with order parameters (*S*^2^) from experimental ^15^N relaxation measurements (Figure 9a,b), as well as other NMR-derived structural parameters, such as ^3^*J*-couplings (Figure 9c) and interatomic NOEs (Figure 9d). These results further validate the enhanced quality of MD simulations when incorporating CS restraints based on NapShift.

## IV Conclusions

Implementing restraints based on experimental data has been shown to greatly improve the quality of biomolecular simulations. Here, we demonstrate that adding a hybrid energy potential based on protein NMR chemical shifts into a molecular mechanics force field can improve the efficiency of both basin-hopping global optimization^16−18^ and molecular dynamics simulations. For each of the proteins explored in this work, identifying a native structure via BH global optimization proved difficult without any experimental restraints. Running BH using an NMR hybrid restraint potential based on the NapShift artificial neural network consistently led to the identification of native-like conformations that displayed the correct secondary structure. This observation held over a wide range of α, the mixing parameter between the original force field energy and the hybrid restraint energy. The best mixing parameter for the systems studied here was α = 0.7. The hybrid restraint potential also proved beneficial when applied to molecular dynamics simulations. Over a 100 ns restrained MD simulation of tryptophan zipper 1, the peptide was able to rearrange from a distorted conformation back to the native structure and remain stable, while an unrestrained simulation started from the same distorted conformation led to unfolding of the β-hairpin. A restrained simulation of ubiquitin showed similar success, as the ensemble of structures generated by the MD simulation closely matched experimental NMR-derived structure parameters.

Our results suggest a number of potential benefits in combining the NapShift restraint potential with existing biomolecular force fields. When inaccuracies in the force field lead to unphysical local minima in the energy landscape, the NapShift potential can reshape the landscape to guide global optimization and MD simulations toward more physically realistic minima. In the case where the global minimum of the force field is not the correct native structure, the hybrid potential can modify the bottom of the energy landscape to favor a native-like global minimum. If high energy barriers prevent efficient sampling of the complete EL, hybrid restraints can reduce these barriers by penalizing non-native conformations, which allows for the sampling of more native-like structures. The hybrid potential can also improve/replace implicit and explicit solvent models, as CS restraints alone can potentially provide enough information on the chemical environment of the biomolecule.

Using a neural network like NapShift to generate predictions for a hybrid potential allows us to continually improve the quality of our model. As more experimental data become available, the NapShift neural network can be retrained to generate increasingly accurate predictions, which in turn will enhance the accuracy of the hybrid restraint potential. We also plan to develop ANN-based hybrid restraint potentials of other experimental NMR quantities, such as residual dipolar couplings, allowing us to further utilize the information provided by NMR experiments. Further studies on larger viral membrane proteins with experimental NMR data have shown promising results and will be the subject of a future report.

## Supporting Information Available

Detailed information on the data used to train the NapShift artificial neural network, including the size of the datasets and the PDB files that comprise the training, testing, and validation sets (PDF)

## Notes

The NapShift method has been implemented in the Python programming language and is freely available at https://github.com/vrettasm/NapShift. A standalone implementation of the CamCoil method has also been released at https://github.com/vrettasm/PyCamcoil, in association with NapShift. The PDB data files and their associated chemical shifts, used for training and testing the methods, were downloaded from https://bmrb.io. The open-source programs GMIN, OPTIM, and PATHSAMPLE are available for download from www-wales.ch.cam.ac.uk. The data that support the findings of this study will also be available for download.

## Footnotes

1 Python machine learning library: https://scikit-learn.org/.

2 Boldface letters indicate vectors or matrices (e.g., **x**), and normal-faced letters indicate scalars (e.g., *b*^(o)^).
