# Supplementary material for: Enhancing Biomolecular Simulations with Hybrid Potentials Incorporating NMR Data
Source: J Chem Theory Comput. 2022 Nov 17;18(12):7733–50. doi: 10.1021/acs.jctc.2c00657 (PMC9753583; doi:10.1021/acs.jctc.2c00657)
Supplement: Supplementary file 1 — ct2c00657_si_001.pdf [file ct2c00657_si_001.pdf]

Supporting Information

Enhancing Biomolecular Simulations With Hybrid Potentials Incorporating NMR Data

Guowei Qi†,[1] Michail D. Vrettas†,[2] Carmen Biancaniello,[2] Maximo Sanz-Hernandez,[3] Conor T. Cafolla,[1] John W. R. Morgan,[1] Yifei Wang,[1] Alfonso De Simone*,[2] and David J. Wales*[1]

[1)] *Department of Chemistry, University of Cambridge, Lensfield Road, Cambridge CB2 1EW, UK*

[2)] *Department of Pharmacy, University of Naples Federico II, 80131 Naples, Italy*

[3)] *Department of Life Sciences, Imperial College London, South Kensington, London SW7 2AZ, UK*

(Dated: 31 October 2022)

† GQ and MDV contributed equally to this work.

* Correspondence to: dw34@cam.ac.uk or alfonso.desimone@unina.it

I. SUPPORTING INFORMATION MATERIAL

Table S1: Training dataset sizes for all six atoms.

Target Atom	Number of tri-peptides
δN	241,501
δC	192,416
$\delta C\alpha$	253,929
$\delta C\beta$	232,944
δH	249,223
$\delta H\alpha$	233,060

Table S2: T-test results between NAPSHIFT and the other methods. Any p-value larger than the 5.0E-02 shows that we cannot reject the null hypothesis that the means of the squared errors are from the same population. Any p-value smaller than the this threshold indicates that the means (RMSD) are significantly different in a statistical sense.

**NapShift**	p-values					
	δN	δC	$\delta C\alpha$	$\delta C\beta$	δH	$\delta H\alpha$
CamShift	4.546E-141	1.347E-15	1.756E-117	1.332E-15	2.639E-69	2.725E-68
ShiftX2	1.423E-08	1.400E-01	4.243E-12	1.428E-25	4.817E-12	5.794E-01
SPARTA+	4.790E-32	1.139E-01	3.138E-12	2.160E-02	8.977E-30	1.339E-02
UCBSHIFT-X	6.204E-02	5.611E-01	3.576E-01	1.428E-01	1.484E-16	2.643E-07

Table S3: The training set includes **2987** PDB files.

2M9A	2LLG	2JUC	2EKH	2I9Y	6Y8W	2MQK	2LZO	2MG2	2K87
2MTE	2D7M	2LFI	2GT3	2L7P	1QA5	1V5M	5IM8	2MZQ	2KVC
5IIR	2LMG	1YWS	5L1C	2JNP	2BYE	5T4R	5SZW	3ZUA	2DAE
2K72	2N24	2CKN	2KSF	2JQG	2LUO	2K2D	2M7T	2DIM	2H3J
1X5I	2KGO	1V86	2M8R	2JN7	2LVG	4A5V	2MYF	6C44	2EQR
2KPN	2DN7	2D89	2EMK	2M6M	2DAM	2JQN	2NXU	2KLC	1UEP
6BP9	2EEB	1X3H	2LWB	2MKH	1UFN	2L8D	5LM0	2DK7	2GL1
2N7Z	2MIX	2MOP	2MBG	2LSU	2YS1	2NLN	2ECY	6QXZ	1X5N
2LFR	2JUO	2KPW	2COM	2KVT	2M4N	2LRD	2MVO	2MW0	5FZV
6NU4	2N1L	2LT8	2LW7	2JXD	2MIZ	2LUU	2MYV	2E9G	2K45
2LH0	2KHM	2RT6	2MNW	5W0Y	2MJD	2EMZ	2MFR	2MV4	2RSW

Continued on next page

Table S3 – *Continued from previous page*

2JV4	2LW8	2LP1	2KD7	2KW0	5ABK	2MLH	2RS8	2EEA	2LLD
2LEO	2AKK	1WFS	2LSM	2DB5	5ZCZ	2L9Z	2K8I	2LX5	2CTL
2EN9	2M6O	2JQE	2RS2	2RSO	6F2X	2LY0	2YSL	2KCQ	6Y07
2KVS	5Z1Y	2JPN	2YZ0	2N4T	2JXT	2DI8	2MHJ	6D74	2LAW
2LW1	1X1M	2JUF	2MZ0	2L6B	2LUA	2LCC	2LQB	2NB2	1V63
2LOU	4AR0	2L7Y	2LE1	2CT7	2I2J	2M0Y	2YSZ	2DLS	2NPU
2MXA	1WGN	5LCI	2YUK	2N86	1X5H	1XFN	2I3E	2LQD	2N49
2LBC	2LW9	5T7Q	2M74	2DML	1WFN	2KCG	2LU6	2M9X	2DHY
2ECV	2E6J	5Z2O	6O6I	2LUV	2JPQ	5ZUH	2NAA	2KZB	2LRX
6NNB	2N0M	6CUI	2L60	2KSG	2K4W	2MPC	5UJ5	2JOD	2RS6
2MTC	6BZK	2KRO	2N7D	2MQ0	2CO9	2N2J	2KL3	2N3T	2FJ6
2LL2	6E5C	2FVN	1ZV6	2JY9	2LIX	2LW3	2EOM	6PQM	1WJL
2DI7	2K10	2JTM	5OQS	2MMP	2HVZ	2M9Y	2RT4	2K49	1WJZ
2P5J	2K19	2L8Y	6OBI	5X3L	1V95	6GW7	2YTM	2MRN	6QAY
5KS6	2MAM	2N9D	4A1M	2DLT	2LA8	2JSX	2K8X	2L50	2M2I
2KDB	1X43	2LRQ	2MHP	5N9V	2EEM	2LOK	2MFV	2RMN	2D90
2MLK	2QMV	1SNL	6MI5	2L3B	2KA7	2N1V	2LPX	5N6R	1DCZ
2G0U	6C41	6LAG	6H5H	2N9V	2LZE	5W3G	2KC9	2MC2	6QEU
2KON	2EMA	2LEZ	5K5F	2JQO	2LR7	2K5G	2RSD	2M70	2RVF
2AIZ	2MUE	2ML2	2MTD	2N7A	2JX2	2MAU	2GJF	2KNI	6BI6
2N9Z	2N1I	2K9P	1Z7P	2KRR	2LRI	2LG6	2FE0	5KTF	2K29
2EOH	2KCU	6O1Q	2LFD	2L1P	5JHI	5K6P	2KZH	2MK6	2LO7
2NDN	1X5B	2N19	2KCO	2L8T	6POJ	2FRW	1X4U	2EON	2MQU
1WIR	2M9W	2KL5	2EMI	2JN6	2XV9	5VNT	2K3C	6AX5	2KPK
5UP1	2HGC	2LRG	1X69	2MW4	2N6J	3ZOB	2LS7	2MVZ	2YSE
2DZK	1UEM	2MDC	2JUA	2DMQ	2MJM	2FYJ	2LSO	2KLQ	1X4K
2DMP	5Z8Q	6SLY	2EMW	2MK2	6H0I	2RT9	2MJ7	2MVG	2DS4
2GTJ	2MHE	2N6N	6DL4	2LL4	2LMI	2K0A	2D7L	1Z65	2COA
2LRT	2YRH	5KI0	6FIP	2N8Y	2LTA	1IUR	2MZU	5GJL	5LGM
2ITA	2EN6	2K8V	2EDZ	2L2Q	2EMH	6F98	1DX7	2L5P	1Z8R
2K6O	2RNG	2MBD	2JR0	2LO0	2KJ9	6TXT	2MWM	2L97	2EGE
2LVC	2KCX	4BHP	2YRZ	2KQ5	2NDP	5OMZ	2K1M	2KX3	2KW7
6GT7	1U89	2B88	2V6Z	5KGQ	2M35	2XK0	6R2X	2DA4	1WXA
2MUA	2JVE	2KOB	2MA2	2K54	2MZJ	2MBC	2LAT	6RC7	6FZK
6FW4	2MQB	2LOE	2KYL	2E7B	1S04	2HH8	2YUB	2N84	2MAW
2MKN	1WH9	2JWG	2LLW	2MA8	2NC9	2HFI	2MAZ	2GQB	2MLG
1LM0	6ER0	6SO0	2L89	2EML	2M7Q	2K8Y	2EOG	6G81	5UKE
2DKM	2N8G	2M1X	2LQ1	2E9H	2CTM	2LRJ	2LXD	2RO5	2M5I
1BQ0	2LK4	3ZG4	2LC3	6D6X	5TTB	2AP7	2JRZ	5N7Y	2DIN

Continued on next page

Table S3 – *Continued from previous page*

1Z5F	2MWH	2MXZ	2LEA	1X4S	2MZV	2RV9	2AI6	2DJN	2LM9
2LUW	1PZQ	2DMD	2K76	5XZK	2GYT	1WXU	2D8T	2MLB	2FS1
2M0N	1WYL	2MQM	2LUT	2NPB	2A9H	5TGW	1WFZ	4A24	2E2F
5X0S	5UE2	2KUD	1V88	2MF7	2MBF	2KYY	2NX6	6FWN	2DAJ
2K5L	2KXV	1WHR	2RML	2KIC	1WGQ	5XJK	2EOK	2KT8	2MVB
5WOT	1WK0	2LYE	2JSN	5SYQ	2KHE	2CT6	2RV1	6MY1	2NB8
2MPF	2DKP	2MYP	2O10	2JWE	1X5Y	2KZ5	5OGU	2KQR	5W8Y
6URP	2ELJ	2JWN	2MQJ	5X1X	2KCT	2LMD	2LRU	2KHK	2MF9
2LL1	1X5D	2LT1	2NWT	1YXR	2L6P	1VEX	2K6I	2L28	2KXY
1WH1	5GAJ	6PQE	2KY9	2EN7	2KKO	2JVW	2LHS	3GRX	2JNK
2MKX	2LK5	2FXP	2KUF	1V87	2JSP	2N4P	6Y06	5JYT	1WYQ
2JN4	2GO9	2NNZ	1WX9	6ALK	5M1U	6MK7	2MWQ	2KZ6	2RSM
2LCV	2L9H	6Q2Z	2MX1	2KYI	2K2C	2OQ3	2P0P	2F65	2M0D
2KT1	2NA8	2E9I	2KJQ	6GC3	5LGF	1BKU	2KE7	2DJ4	1X4I
6MK4	2L81	2LPV	2L1O	1WJP	2M1I	2JPD	2YS3	5VO7	2MHY
2KZV	2M30	2MOG	2FEK	2N3L	1YUT	2M3N	2YUA	2M83	2M2A
5K57	2JTY	6YI3	2ECG	2YON	2CT1	2IVW	2K32	2KI3	2M72
2JYO	2K4Z	2L84	6FI7	2LPK	2CTK	2LAK	2MEU	2MQE	2K1G
2YTE	5IQ5	2KK7	2JRO	2KT6	2N6R	2MEY	2M68	2LY8	2K7R
2KK1	2K2F	2KUC	2LKB	2KAV	2LJX	2LJK	2KCW	2N40	5O7J
2M29	2MJ6	1X6D	2EKJ	2KBZ	1V5S	2LC1	5AJ1	2KAJ	2MPL
2KU3	2ZAJ	2KNZ	2L25	2MOX	2N5M	2LN4	5WOX	6PRQ	2MU8
2KCA	6SY2	2LM2	2KSR	2KSW	2L09	1WY8	2N12	2M97	2EN2
2D9V	2DBM	2LCM	2EC7	2MPE	2ML3	2NA2	4CH0	2DZL	6COQ
5ZNU	2KMG	2MCA	2MMO	2BIC	6RFM	6I9H	2KPP	2RPS	1WEN
2LEQ	6O8J	1WJN	2EOJ	2LAH	2RNJ	1X05	6VFO	5WUZ	2MPQ
2M0R	2MB7	1WJV	2K5C	2N68	1YZC	5AGQ	2LLA	2LQ6	2JOB
2HC5	2MI1	2MG4	2NC2	2JOX	1WK1	2DJB	6BQI	2CU7	2ND2
2KLU	2MRL	2MXW	2YU0	2M6Y	5Y0I	2KY4	2HG7	2JR2	2MOF
2MJU	2MUJ	6DMZ	2LMA	2L3A	2KSD	6MZA	6O3S	2LVH	2M73
2LJB	2MHS	2KY5	2LY3	2JR1	1BCI	2L2D	2YTJ	1WI8	2K7N
2MD6	2K8E	1RG6	2LAM	2N65	6FD7	5KQB	2L9P	2LJA	2EMP
6HVK	2LZN	5GWG	2N99	2L7K	2DJ2	2K85	2KL2	2ARF	2L04
1UJS	2RPZ	1VA9	2EP0	2MI5	2DMK	2B1U	2RVC	6VTI	2LX4
6UZJ	2M2K	5T42	2KLZ	2M5L	2MEK	2K4V	3ZBE	2KRX	5MTI
2CT0	2KB6	2CTJ	2M5H	2JRL	2EDB	2KKV	1Y7X	2K0L	2JXY
5TVZ	6R3C	6FBL	5ZAZ	1WJJ	2D88	1X5Z	5W9F	2LV5	2KTL
6AMW	2EME	2KCP	1WH8	2RTS	6F46	2N6B	2LYV	2DLM	2JR8
2YUM	2LLX	5LW4	2JRR	2LOB	2MG1	2MDV	6TIQ	2MI2	2BL6

Continued on next page

2EOU	2KN8	2JNU	2N90	2ECM	2MI6	2MJK	2YTK	2DA5	5XDI
2KL6	2MLD	2DMC	2KLX	6Y1H	2N10	2NAE	2N2A	1UGV	5O2Y
2M0T	6VEE	2K9Y	2MPK	2LAI	2K8Q	2NO8	1O1W	2K5F	2K2O
2YSM	3ZPM	2M2Q	2M9U	2MVF	2JOP	1UJU	2MOU	2JOE	2MF8
2HWT	2DMS	6F55	2K57	2KPI	2K4B	2ED7	2DA1	5NR5	2KJ8
2JN0	2MA4	2EDY	2MHK	2LSS	1TUJ	2JS7	1WJI	6AX2	2L3T
2N34	2N3F	2JWS	2RT3	6O0C	6QKF	4AXP	1BA9	2RV8	2LXH
1XPA	5O2Q	2K75	2KKY	4BIT	2GZP	2OT2	2RUF	2LZ5	1WJT
5JPW	2KR1	2MDG	6MPO	2LUL	2KX0	2NB9	1WHN	2LJ8	2LM1
2MO1	2N6E	2MD1	2M1J	5JI4	2F1E	6MBM	2KF2	2MH9	2D9U
2KYR	2MYI	1WYN	2CO8	1WH3	2DZJ	2KCZ	2K8A	2JVD	2AVG
6RQS	2L1T	1LR1	6DO6	2L4D	2OSR	2RM4	1V9W	2KHV	2RPB
2NBP	2N2C	5U6L	2L7F	2LZ0	2MUI	2G7J	2FI2	2M5Y	2L6N
5UJH	6U3R	2G0Q	1ZKH	2N5G	5LXL	2NCS	2JYA	2N1S	1X44
2MI7	2JR5	5NMY	2MNY	2JNY	2AQE	2MXX	2L7E	2LNT	2L9G
5WQZ	2RUY	5MOU	2JWH	5KVP	2LF2	2N03	5AHT	2LWX	2JNG
2L4V	2MF3	2N21	6CKQ	1WI0	2LXI	2MLO	2EOF	2DL3	2KZX
2M47	2DII	2JMS	6HQ1	5GO0	6AP5	2MWI	2KHZ	1M3G	2YS2
2I3B	2LW6	2LX0	1WGY	6GSE	2KUT	5JOJ	2DMU	2LA2	5M1H
1WYP	2KYS	2KX2	2D8S	2G2B	2LLI	5M9U	2KIV	2I1P	2KJG
1HYI	2LQO	2N2T	2MPH	2B1W	2YHH	2NBM	2EPS	1WGS	2ERS
2M64	6QH2	2LB6	6EWS	2MW8	5Z9C	2LNX	2MSY	4D4W	2ENA
2DJR	2EM4	6CKV	2D7Q	2JW8	2L76	5X3Z	2M4G	2H1Z	2EEC
2MZC	2L4W	2MUB	2AYX	2D8V	2KQ2	6NAN	2IZ4	2RO0	2LWS
6V6T	2JT1	2KGT	2N6V	1ZRR	2DO8	2ECT	2JS3	2YTT	5UI6
2LXR	2K0Y	2JPO	6MG9	1NR3	2L9U	2KSV	2I1D	1X5L	2M08
6CEJ	2KVO	2JXP	2DMN	2MMY	2ML8	2COF	2L2N	2LB0	2MU7
2DHX	2NBH	2D85	1WJ1	5XBD	2MT9	2KJK	6Q6E	2JMO	2N9G
2KN1	1X3C	5IX9	1X3D	2NBG	2E5K	6SGO	2L92	2LXU	2E7H
6H0Q	2GZO	2IKD	1X5J	2YRJ	2LCH	2NX7	2L02	2LE4	2EE0
2N2Q	2KZY	2JS1	2FHM	2MM3	1WFT	2D92	2YUS	2YUN	2I9O
6PRP	6CEI	2CUD	2KK2	5ZOR	6MIE	2M7A	2ML6	5WOY	5XIV
2KP6	2MV2	2GDT	2AN7	2D9X	2LLZ	2N81	2IJY	2MXC	6C00
2KSH	3NLA	2GRI	1U3O	2MV6	2DJU	2LR3	2JRA	2MHV	6BUC
4B8T	2JQ4	2KPJ	2LDF	1UJX	2LTU	2K1S	2MN6	5VJ8	2H25
2G5M	5UTG	5M4T	2DA6	2LDS	1X5Q	6BV7	1WZ6	2L9N	2L4F
2MZ8	2RR8	2DJ3	2M3V	2MVJ	6OTA	2NDI	2GQE	1X5G	2KZC
2JZC	1L7B	2KIF	2DJ9	1ZLC	5SXY	2MRC	2LFU	5W3N	2GJ0
6HD2	2KCH	2DLU	6OHX	2DK4	6TG5	2KVV	2N5L	2FGX	2JXX

Continued on next page

Table S3 – *Continued from previous page*

2L7W	2L6M	1JI9	2M4Y	2MY6	2M3L	1X32	1X6E	2KJV	2MJ8
2N8N	6CAH	2M19	2AJ0	1UH6	2M0S	2M7P	2MXO	2KM6	1WJU
2LBN	5VWE	2L57	2EOS	6NK9	1WLN	2JSO	2M07	2DBJ	2K4Y
2N72	2LNE	2LXN	6G8O	2LPM	2MR9	2JZ8	6CKF	2K9Z	2LYC
2KBV	2N5Z	2MXD	2K2E	2BZT	2LLL	2L7R	2MZZ	1WXT	2ND4
2ECL	2K1E	2RS4	2JNT	5AAY	2JQ3	2EP2	2KNR	5H7U	2M26
2M3S	2L0D	3ZTG	2KTC	2LS4	1V5P	1WFU	2LKN	2K5D	2MPV
2APN	2MUP	5TCE	2MDR	2D8H	2JO7	2RNZ	2LE9	2D9Z	2KRU
2L0W	2DHJ	2N7Q	2ML1	2L8V	5KES	2NVJ	5O1T	2JPI	2GRG
2PLD	2WCY	2JMU	5FRG	2IKE	2KFP	2K31	2L73	2RU1	2KCK
2DJS	6QK5	2L43	2L4N	2LWF	2N8B	2K2X	2HTF	2LVN	2ENC
2L5R	2M6R	2N3S	2JXU	6B3N	2MYG	2N98	2LJ9	2NBB	2L31
2LNV	2EM9	2KRF	2LTP	2V9H	2EDX	2KWL	2M9M	5VFW	6BA3
2ASY	2LWL	2N4O	2LPC	2RRK	2M3W	2LNJ	2D8U	2YRP	2HDL
2M7X	2LJS	2LX9	2L2R	2M7L	2DMF	1V5K	2LCE	2ECN	2KKZ
6UCH	2LJM	2LGR	2CSY	2NAW	6E9M	1X5R	2KE4	2MTL	6MK5
1UJO	2DIZ	5V4U	2RUX	2M3I	2AYA	2MK5	2KA5	5MGQ	2LS8
2MKW	2MZR	1R79	6D53	6E4J	2KPU	2LT9	2KZA	2LIY	2M63
6RH5	2MM6	2JS4	1GEA	6KR8	2FHO	1J3T	2L3M	2DAW	2LXO
5WLP	2M9H	1QP6	2M1S	2KGG	2MM8	2K9I	2JUB	2YUP	2L48
2M05	1V31	6NE8	2DT6	5YQ3	1FLS	2JXW	1A5J	2LQ9	2LNU
2E7C	2MTI	2LIE	2N8F	5LQV	2L8O	6XXU	2CUF	2KOE	2KO1
2MC3	1UE9	1WHU	2JM2	2KQ1	2LU7	2MHG	5MXL	2K5P	2OV6
6AHZ	2M0M	2NAV	2FRY	2EPP	2KN7	6HZ2	2IN2	2XA6	2M32
2KKM	2AVX	2DMM	2MDZ	2LJP	5LBJ	2LXB	2H7T	1WJS	2KJ5
2N59	2LS0	6PPC	5UNK	6RPV	5O2V	6CJZ	1N27	2L9J	2L8A
2ND7	2KC6	2M3K	2LOS	5OAP	2MM9	5UZL	2MNS	2RRI	2MN4
2N57	2LFJ	5ION	2LKQ	2EM5	5VSO	1UG7	1Z7T	5XM4	2KVR
6HNF	2JOV	2LC5	2DJ0	2LVL	1X6B	2K6N	2GUT	2LLY	1WG5
6CL3	2LQN	2D8J	2MPW	2M7O	2LFH	1X5W	1WX7	5JWJ	2DL8
6BQS	1WZ4	2COT	2MZS	2LXE	5JPX	5ZMB	2MLW	2CSP	2MU3
2B89	2KXI	2MT4	2LV9	2KD2	1Z1Z	1X5K	2MRM	2LOL	6HH0
2N1R	2NAQ	2MBY	6SOE	5VWL	2MPO	2MYY	5L82	6EKA	2N5K
2M85	2MHC	2KRA	1X45	1CL4	5JYH	2KCJ	2M9L	2NAB	1WF5
2M0Q	2LGP	2NAJ	2I5O	2N3D	1X1F	2LCU	5LVY	2DA0	2LVF
5HUZ	2KYC	2KTS	2LNY	2M2U	2FE9	5UP5	2MMM	1VDI	5J6W
6YTS	2L62	2KKC	6I2O	2KXD	2DOG	2Z4F	2M09	6TOB	2X8N
2H7A	2LC0	2YTG	2LN7	2LRA	2K4J	6MJV	2L6A	2GOW	2HGA
2L9C	2MH1	2FWU	2K37	2ND9	6PX8	2LX7	2LHN	2JWT	2M0W

Continued on next page

Table S3 – *Continued from previous page*

2BYF	2KWT	5FIM	5MMU	6RK3	2DM8	2N3J	2KUY	2N2Y	2K6Z
2L6R	2MAH	2Y4W	2LRC	2MSO	2K9O	2K5R	5T43	1WH7	2L9L
2CZN	2KJP	2KO8	2D9W	5U9B	6PVT	1WIK	6NUG	1WGR	2LKI
2MKS	2KD1	5YI4	1UFG	5WDZ	2DKQ	2FVT	1L6U	1WFO	2EOP
2G1E	2LUC	5UOI	2YTF	2LOQ	2LEP	2N88	2MHD	2M65	2LTJ
2L33	2K27	2EOW	2JU5	2MY9	2LOJ	2NCA	2LYI	2M5V	1WF8
2JGX	2DJT	2KLL	2I7K	1WJR	2K7S	2NSW	2D7N	2RN9	2KWZ
2RRF	2KLY	2EE7	2RN7	2MTG	2LF4	2N8X	2KZ9	2N3P	2YSP
5J17	2EM1	2LSW	2YOM	2N05	2K9S	6G5S	2CXJ	1UL7	2LU3
6CZT	2N85	2M7B	5N9Q	6PX7	2K7B	5WXE	1V5Q	1UM7	2I4K
5FRH	2MZY	2L8B	2N2F	2LJW	2MXN	6V5L	2N7F	2BAF	2LC6
2KXA	1WI7	3ZEH	1X3B	2LZL	2PNG	2HFD	2KAT	6Q1X	2O3D
2KM4	2LUG	2LX6	2KDP	2CTD	2N8I	2POA	2KZK	1WJK	5US5
2JR3	2JVF	5ZMR	2LPD	2MQN	2D7P	2N11	2LGZ	2L06	2K28
2ELL	2LDU	2M2F	2DI9	1WF7	2LTH	2KKR	6QBL	2KKE	2MP1
2MJ4	2MDU	1ZR7	2NDD	2K1H	2KFB	2JRH	2M6K	2JVA	2RUW
2EQI	2MI9	6BB6	1QFD	2M3X	2JPF	2K3J	6D6S	5OQK	2LRV
4C7Q	2EA6	2KD0	2LQM	2N1N	2KZN	2JQQ	2NAR	2EHR	2JZ4
2ECZ	2M4V	2G0K	6TR8	2MZB	2EE8	2MT7	2M3C	2MX0	2PAC
5UI7	2KMV	2DMT	2KN0	2MIC	6GMS	2NCP	2KFL	5MWW	2RVA
2N26	5UYO	2EN0	2K7I	2LTM	1UEW	2K6D	6OCV	2HJJ	5LG9
1ILY	2DIA	2JX8	5YDX	2LNA	5T3Y	2LYY	2DAZ	2B3A	2LE3
2N6G	5M0A	6BZJ	2ND3	1V5U	5YZ9	1V5R	2L47	2RRS	5YXI
5ZB6	2MOT	6DST	2EOX	6GQ9	5MS9	2YSO	2N5Q	5OUN	2MU9
5O6F	2KIW	2KG4	5XND	2YUR	2K3B	1XHJ	2YSQ	2LTL	2LIZ
2GO0	2MC8	1X5X	2ROH	2I83	2M1W	2MYX	2KTA	5IZB	2DIB
2DAX	2DHI	2J0Z	5LO2	2D87	2MRD	2DAH	6NX4	2K24	2KMT
2O4E	1SXD	2A63	2LQG	2N4B	5KK9	2EN4	2L9V	2LIO	6KLM
2KK6	2M46	2HGK	1K81	2DAL	2K3R	2RSX	2LS2	1XQ8	2DIG
2CUE	2KES	2QL0	6GSF	2N17	2LCJ	2L4H	2N71	1WGW	2MTX
2K2A	2MU6	5H2S	2RSG	2MZT	2KQ8	2JZ6	2K4X	2MO5	5NQ4
4UZM	2MV3	2LB7	2LN8	2COD	2M71	2IDA	2C06	2M7N	2MP4
5J8T	2DJC	2JUW	1JSA	2N7G	2MMB	2N8W	2MQS	2LOP	2NPR
5ODD	2DL7	2L9M	2RV5	2MDL	2N30	1WFI	2N6O	6XWI	2M45
2BTT	2M6N	2LPF	1XOX	4APD	2KA0	6F8E	2KEP	1WES	2K6A
2DJ1	2JPE	2LMB	2LGW	2KDD	6FTK	2MBT	2LHK	2MBX	2EDE
5GVQ	2L5C	5T7C	6VED	2L52	2DAV	6UHW	2KCL	2LF0	2LGV
2MOK	2KI8	2LC9	6MZT	2MG9	1WIE	2KVA	2JOO	2LF6	1UFX
6OBK	2DLP	6MW6	2MWX	2KHQ	6R0J	2EMJ	2KPA	1TVM	2MH4

Continued on next page

Table S3 – *Continued from previous page*

2YUU	5H3N	2RV6	5Y70	2N62	2DZI	2BN8	2RQL	2M5T	2LSE
2KIG	2RS7	2L6K	6BZL	1UIV	2N5B	2MKJ	2JS5	2N92	2LQ0
2DA3	6I3R	2LOM	5XQM	2MWG	2KC7	5WAH	6F3K	1B3I	2NCY
2N3Z	2LFG	2LZJ	2MQH	2EEL	2JZA	2JSW	2A3J	2K02	2DJK
2MHW	2MTW	6GWM	2LXL	2YUO	2ITH	2LON	2LOY	2D86	2JXF
2MT8	2M52	2LJ7	2NC8	2N6U	2M80	2LCR	2M6U	2N5S	3ZQD
1WGH	1UJV	2JY0	5UHU	2KA1	2MTY	2MM5	2MFK	2K6S	2L6O
2K52	2NBS	2LQR	2LA3	2MA1	2MUU	2KYB	4BA8	2DME	2DMB
2KT7	2I7U	2MDQ	2KVH	2L95	2LY5	2JP2	6PIN	2KY3	2KHU
2E6Q	2MXQ	2JRT	2RU9	2E7K	2H0P	2M01	2AL3	2M8H	1X1G
2EE2	2MXM	2LW4	2KWC	2N5D	2YRM	2KRN	2M76	2L3F	5M9Z
2DAG	2MXE	5JTK	2KHN	2JNZ	2MMG	2DOC	2LE2	6G7G	2MNQ
2KPQ	5U87	2MM4	2MDK	2JO1	2LC2	2MRW	2MGW	2LLE	2LVW
2N00	2E9K	2JQW	4UZW	2M87	6POR	5O57	2JS0	2MPU	2MKZ
5YFG	2K73	2K5H	2EMF	2JMR	2RSC	2FFW	5KIZ	2L8K	2NCE
2LD3	2M2L	5T82	2ARW	5AIW	2NBA	2LVR	2KZW	1JI8	2KPY
2MYW	2YTD	1AXH	2LV2	2L1N	6EHZ	5WOE	2J48	2L77	2LJ6
2RUZ	6NL3	5JS8	1V5O	1UHT	2K6V	2DJM	1ND9	2G7H	2LS3
2MFL	1WIN	1WGK	2KRK	2KI9	2MRA	2MR3	6O8S	2LUS	5XDJ
2V37	2NDC	2YUL	2LBA	5OBN	2N31	5WOD	2LSA	6U6G	2LS9
2MJC	1V32	2JUH	2E6P	2KWP	2RV0	5ZPV	2MYH	2L3N	2KMS
1J26	2KT0	2MGY	2KQK	2EOL	2MOQ	2KLB	2K5V	2L22	5MN3
2MCR	2RUH	4BF8	2KKN	2N6D	2GW6	2KJW	2LEK	5XME	2CSZ
2EMG	2MGQ	6SZC	1BR0	2MLU	5GVO	5T17	5WE3	2MK4	2N9O
5T6V	2M4L	5KZO	2E0G	2MJZ	2ECC	2N5F	4CSQ	1WF1	1WGV
2KC0	2KQP	2LQ5	1ZG2	2KLE	2ED0	2RV7	2JZ2	2PCO	2MDT
1WF2	2E5E	2N8S	2MU4	6EQY	2LLH	2LXF	2YS4	6FFQ	1V9V
2MXB	2ED8	2JN3	6CGX	2DBA	4B6U	2MFA	6CPK	2DHS	2G9L
2KIT	2LCL	2MR6	5Z8I	2NDB	2L05	4C26	2EFI	2K0Z	2KUM
1VA8	2DZM	2NDA	6MIF	2EE9	2G31	2L9R	2JTV	1X4L	6Q5Z
2KK8	6SJX	2EWL	2LXW	2YTH	2K1K	6E4H	1JJJ	2GX1	2MPJ
2MN3	6PMG	6MM4	2EM2	2L4B	2FY9	5B7J	1WEZ	6CJD	2LSN
2GJI	2LKW	2AIH	5MSL	2CU8	2LBX	2MIQ	2CSQ	2LQ4	2D8R
2H80	2NCH	2VCD	5LME	2MSF	6BX9	6CMY	2M6J	2LGH	2RV2
2KN6	6GRV	1V6G	2COO	2LZY	2KIE	6E26	2LR9	2MY1	2KUS
2EXD	5MMC	2MJ3	2MDW	2EOZ	2I50	2LHJ	2KAK	6FE6	2COC
2MUD	2CKA	2LY7	2KB4	4B2V	2KXX	2KMW	2KG2	2EOI	2MX7
2K5W	2LGJ	2KNG	2LJU	2E7M	2RTT	2LI6	2LO2	2N52	2K5J
2N0X	2L08	2KUE	1V6E	2GJY	2FQA	2LV7	5NBB	1WFJ	2E6I

Continued on next page

Table S3 – *Continued from previous page*

2N6F	2LXK	1WH4	2M51	2JNS	2E63	2LS5	2JQV	2I1T	2LEW
5OAY	6U4M	2DN6	2MTF	2E0H	2L9Y	2N5U	2L82	2MIU	2AGH
5NZ9	5LFI	2AJE	2JQ8	2KBB	1X6F	5IEC	2LEN	2NA0	2NCN
6BF2	2LAA	2MBS	2LDE	2EM3	2L5Q	2M48	2LDY	5KPE	2LTR
2L10	2DL4	2MVA	6V88	2D8Q	6MSP	4A53	1WH5	5VAV	2LE7
2JT0	2CSW	2LWQ	2KZ4	2FFK	2DF0	2ECI	2OA4	2JMM	2NB5
2EBM	2EEF	2MX2	2LJ0	5TTT	2E61	2KAE	2DI0	6H0J	5X34
5VKG	2KOJ	1Z8S	2MQL	2RQ0	6CPI	2W9U	5ML1	2KZ0	2KZQ
6IB6	6ALY	2LRH	2DIC	2ELK	2EED	2MHF	2YRL	2DAF	2M1T
2LT2	2M5F	1J0T	3ZPD	2G1D	2MHH	2EMC	5U3H	2MTS	2K0Q
2LGY	2GPF	5NF8	1EH2	2EMM	2KEN	2V1N	2JOY	2L9F	6TH8
2M9K	2MQ8	2N64	2B7E	2KKQ	2NAS	2DHK	2KFS	2L7B	5Y4B
2DQ5	2KO6	2DL9	1Z2Q	2N5J	2LWT	2L8S	1WJ3	3ZJ2	2WH9
2MC4	2LFV	2D9T	2M3D	2N51	2KKL	2M5R	1T2Y	2K48	2LQL
2KDN	2M4I	6GO0	2N93	2M3E	2JO6	2MH8	5A4G	2K0M	2RMY
2N4D	2KVU	2HDM	2LNM	2MX8	5IJ4	2K4M	6NOM	2LP7	6QS0
2RRN	5JPL	2KCR	2KRM	2M1L	2M7E	5I8N	2MIO	2ED1	1WFG
2MUK	2PXG	2MAB	2GGR	2L6U	2LF3	1WFW	2HFQ	2M1B	5JYU
6R8D	2M4F	2YS9	1X5A	2LQU	5VR5	2RUV	2K50	2K2M	5U9S
2RU8	1WHA	2DIL	2GZU	2JY8	2DL5	2F09	2L69	2YS0	2KNX
5I1X	2NBV	1X4Z	2RTX	2M5J	2JML	2LKZ	6GGZ	6GIG	2LFC
2CT5	1SRZ	2NCG	2M1H	6GNZ	2L7Z	2MHN	2K0D	2EBK	2EGA
6G03	2DWV	2KJL	2LST	5LCS	2LKD	2YT2	6OQH	2L5T	2EGC
5I4G	4UZX	6Q44	2N2E	2DEF	2KQM	2UVS	2MZG	2MEW	2RST
2JU7	6GBD	2MME	2MCZ	2MAO	4CRP	1WJO	1WFY	2LF8	2ABY
2EOY	2LRO	6EFE	1WXM	6CCJ	5ZFO	5UTV	5MFY	2BBU	2LM3
2K5I	6GFT	2GMG	1WIL	2CUM	2JN8	2CSI	2KIZ	4B2S	1X4X
2DA7	2RSQ	5KJG	1N89	2MQA	5X5S	2DVH	2YQL	1PD7	2NDH
1PPX	2HJ8	6GS9	2RQ1	2RVH	5IEB	2KZR	2KPM	2KV4	6SAI
2KT3	2KOK	2K6B	2MYJ	2KKJ	2HEP	2MRJ	2N1W	2NCV	2L4O
1W7D	2LQ2	5JZR	1V5N	2KAM	2AKL	6EWT	2LVA	2LCK	2KVZ
2LNZ	1X6G	2CTO	2K16	5AAQ	2ECW	2N1P	6MF8	2DIY	5Z26
2RUR	2N9W	2DMX	2LZX	2EE3	6AXD	5L3N	1WH6	1X4Y	2LA7
2KK4	1J0F	2LMR	2K1B	2L4U	1WJQ	2MKE	2L93	2LVS	2N2Z
2N18	2LSI	5KQJ	5IAZ	4CYK	2MMH	2LZU	1X5F	2MW1	1UFF
6O3Q	2MZ7	1S8K	2OFN	2M4E	2LQJ	1Y58	2DLG	2MQ1	2KV9
2JSZ	2KGK	2RPJ	2KT9	2ML5	6CWS	2LZ1	5MQX	5LWC	2KW8
2DKT	2RVK	2KFD	2RV3	2KJZ	6CPJ	2MS3	2KC5	2KMU	2LFE
2MD9	2L0C	2LR4	1X5M	2MHL	4B19	1IYM	6BTV	2MNI	2KNU

Continued on next page

Table S3 – *Continued from previous page*

2LLK	2DHZ	2MZ6	5TJ1	6QWR	1UJT	2KPT	2JWY	2MT5	2JQZ
2G9O	5WLX	2RVJ	2K43	5KPH	1ZXF	1UJY	2DJA	2LK2	2LNI
2MUQ	2JZY	2AMN	2I85	2MUF	2JQ6	6GS5	2EN3	2EN8	2KYA
1TM6	2LRS	2EMB	2MD0	2GZY	2JS2	2K8D	2RUO	2E8J	2EKI
2LJI	2N3E	2LVB	2LCX	2LD1	2L7J	2I2H	1EJQ	2YUE	2A7Y
2LQ7	1WWY	2MW7	2MIT	2N9U	1VAE	2NB6	2E34	2KPO	2LO3
2XKS	1NY8	2PP4	6C2U	2EOR	5WCV	1WEY	2KPH	6NW8	1WFV
2LCW	2N5N	5XN4	2LK1	2M1C	2LHT	2DA2	2M5X	2RRD	2LAU
5VTO	2M2E	2LQ8	2M4H	6I57	2KRG	2LHU	2LSQ	2KEO	2CTE
2JM5	2KGS	6QBI	2JOZ	5LXK	2JSA	2MA5	2N47	2N35	2E9J
2MK3	2EM7	2MIM	2MUN	2M6A	1ZJQ	2L7N	2DJV	2LVJ	5W8Z
2LWU	2N2R	5TN0	2N8K	1DGQ	2E45	1WFM	2BBX	2NBR	2M0C
5JN6	2KYW	2MUY	2N7E	2M9V	2M9I	2M1U	2KV7	2M7Z	2L1S
2RU5	2JR7	2KL7	4AAI	2GVS	2LUY	2LQ3	2L7Q	5AAZ	1WX8
2L5O	2MXV	2LDK	2LCQ	2LBO	2OI3	2KRT	–	–	–

Table S4: The test set of **250** PDB files. The examples that were used for testing the performance of NapShift against the other four methods, were randomly sampled from the original dataset and were excluded from the training phase.

2H3K	2LA4	2LWP	2N29	2NAU	5IRD	2K4Q	2YUQ	2KOZ	2N2U
2LGO	2L80	2EPQ	2HJQ	4BMF	2JRB	2KAY	4AKA	1QX9	2FO8
1X6H	6NUI	2RRE	2LR8	2LGL	2E29	6EZ4	5ID3	3ZJ1	2MS7
2MGV	1OVQ	2KP5	2LBB	2N4F	2AYM	1Z66	2FQH	2MKL	2M2J
2AGM	2LVX	2LPU	2YRQ	2MD8	2KHR	2M7H	1TVC	1WF9	2RRL
2K5E	2H41	2DMZ	2LG4	2KZS	2MBL	2JUG	2EM6	2CTF	2NPL
2CT2	2K4N	2N1K	2MLJ	2EPR	2AMI	2MF2	2IUE	2JRS	2JNH
2MUX	2LJH	2LUZ	5ZCN	2MQD	2KQ9	2K3D	2K2J	2DB8	2MT3
2EDD	2MCF	5XI9	2MGS	2JOQ	2LIU	2LN3	2JX5	2MK9	2EMV
2NCL	2MCY	2KOU	2N41	2CSS	5IE8	2EE6	6QBZ	2M5W	2LCP
6F24	2KOY	2EOQ	2LNB	2OSQ	2I59	4CZ4	2ML7	6DHR	1UWO
2MU0	2M8U	2MBE	2HI6	2ECJ	6TR0	5LKN	2N39	2EOV	2LRN
2EA5	1I17	2MTM	2LR6	6NVZ	2LQT	2LWJ	2NAN	2LLV	2ENH
2EMY	6F99	6CGH	5IX5	4CPG	2N7P	2LM4	2M8C	2L83	2MP8
2LV3	2KL1	5NB9	6HKC	2KR7	2LTO	2KEL	2M9G	5T3M	2LUQ
2KDR	2KXE	2KKU	2M2B	2L5L	2M4M	1WYO	2MII	2JRP	1WFQ

Continued on next page

Table S4 – *Continued from previous page*

2EE1	2A3S	1COK	2KHA	2EM8	2KK0	1X5C	2LS1	2EE5	6N8C
2LDI	2MA6	1WGL	2LKV	2LOR	2CH0	2KKS	2LTD	2JUZ	2MA7
2MVT	2LG1	2LN0	2K3I	6HT4	1X3A	2NAY	2LGX	2LYH	2LKY
2D7O	2MUG	2MN2	1WYR	2KUN	2K3G	2K4E	2EMX	3ZGK	2M4K
2JZ5	2L01	2KHC	2LME	1HZL	5NCE	2KB9	2MQC	2KNA	2LD4
2LKL	2KRS	6O6W	2CT4	2LTE	2JQ5	2KJF	1RJH	2LYX	6GWX
2LHR	2K9N	2K06	5NR6	2JPU	1X5E	2KER	2KGJ	2KIJ	2JMP
1WJW	2MMV	5MXT	2MMW	6C8U	1Y4E	2D8I	2L9D	2YRY	2KEY
2JRF	1WGG	2KW6	5LAH	2YR3	2N78	1WIM	5TMX	2M36	2KZ3
